# LncRNA-XR_002792574.1-mediated ceRNA network reveals potential biomarkers in myopia-induced retinal ganglion cell damage

**DOI:** 10.1186/s12967-023-04662-x

**Published:** 2023-11-06

**Authors:** Xuejun Wang, Qinghong Lin, Shengtao Liu, Xiaoying Li, Xiehe Kong, Yuliang Wang, Weijung Ten, Yangyi Huang, Yanting Yang, Jing Zhao, Xiaopeng Ma, Xingtao Zhou

**Affiliations:** 1 Eye Institute and Department of Ophthalmology, Eye & ENT Hospital, Fudan University, Shanghai, China; 2 NHC Key Laboratory of Myopia (Fudan University), Key Laboratory of Myopia, Chinese Academy of Medical Sciences, Shanghai, China; 3 Shanghai Research Center of Ophthalmology and Optometry, Shanghai, China; 4 Yueyang Hospital of Integrated Traditional Chinese and Western Medicine, Shanghai University of Traditional Chinese Medicine, Shanghai, China; 5 Shanghai Research Institute of Acupuncture and Meridian, Shanghai University of Traditional Chinese Medicine, Shanghai, China

**Keywords:** lncRNA, Myopia, Retinal ganglion cell, ceRNA, lncRNA-XR_002792574.1/miR-760-3p/Adcy1 axis

## Abstract

**Background:**

Long noncoding RNAs (lncRNAs) play a key role in the occurrence and progression of myopia. However, the function of lncRNAs in retinal ganglion cells (RGCs) in the pathogenesis of myopia is still unknown. The aim of our study was to explore the lncRNA-mediated competing endogenous RNA (ceRNA) network in RGCs during the development of myopia.

**Methods:**

RNA sequencing was performed to analyze lncRNA and mRNA expression profiles in RGCs between guinea pigs with form-deprived myopia (FDM) and normal control guinea pigs, and related ceRNA networks were constructed. Then, potentially important genes in ceRNA networks were verified by qRT‒PCR, and Gene Ontology (GO) and Kyoto Encyclopedia of Genes and Genomes (KEGG) pathway enrichment analyses were performed to explore biological functions in the RGCs of FDM guinea pigs. The important genes and related signaling pathways were further verified by qRT‒PCR, immunohistochemistry, immunofluorescence and Western blot in myopia in FDM guinea pigs, FDM mice, and highly myopic adults.

**Results:**

The distribution of RGCs was uneven, the number of RGCs was decreased, and RGC apoptosis was increased in FDM guinea pigs. In total, 873 lncRNAs and 2480 mRNAs were determined to be differentially expressed genes in RGCs from normal control and FDM guinea pigs. Via lncRNA-mediated ceRNA network construction and PCR verification, we found that lncRNA-XR_002792574.1 may be involved in the development of myopia through the miR-760-3p/Adcy1 pathway in RGCs. Further verification in FDM guinea pigs, FDM mice, and highly myopic adults demonstrated that the lncRNA-XR_002792574.1/miR-760-3p/Adcy1 axis in RGCs might be related to cGMP/PKG, the apelin signaling pathway and scleral remodeling.

**Conclusion:**

We demonstrated that the lncRNA-XR_002792574.1/miR-760-3p/Adcy1 axis in RGCs might be related to myopia. On the one hand, the lncRNA-XR_002792574.1/miR-760-3p/Adcy1 axis might inhibit the cGMP/PKG and apelin signaling pathways in RGCs, thereby causing RGC damage in myopia. On the other hand, the lncRNA-XR_002792574.1/miR-760-3p/Adcy1 axis may cause myopic scleral remodeling through the ERK-MMP-2 pathway. These findings may reveal novel potential targets in myopia and provide reference value for exploration and development of gene editing therapeutics for hereditary myopia.

**Supplementary Information:**

The online version contains supplementary material available at 10.1186/s12967-023-04662-x.

## Introduction

Myopia is a worldwide public health problem that typically starts in childhood [1]. During the COVID-19 pandemic, increased near home learning and reduced outdoor activities increased the risk of myopia in children and adolescents [2, 3]. It is estimated that the number of myopes will increase to 4758 million (49.8% of the world population) by 2050 [4]. Myopia is caused by a combination of environmental and genetic risk factors. In recent years, a growing number of researchers have focused on the genetic pathogenesis of myopia [5, 6].

Long noncoding RNAs (lncRNAs) are a class of transcripts containing more than 200 nucleotides [7]. Characterization of lncRNAs has revealed their functional roles in regulating different cellular processes. These include pluripotency maintenance, carcinogenesis, lineage commitment and pathogenesis of various diseases. The functions of most of the over 173,000 discovered lncRNAs are still unknown [8]. LncRNAs can mechanically act as competitive endogenous RNAs (ceRNAs), which competitively bind with miRNAs to reduce miRNA regulation of their target mRNAs [9]. Previous studies have shown that lncRNAs are associated with a variety of eye diseases, including glaucoma [10], age-related cataracts [11], diabetes retinopathy [12], and choroidal neovascularization [13].

Recent studies have shown that lncRNAs play a crucial role in the pathogenesis of myopia. For example, the abnormal expression profiles of lncRNAs in the retinas of form-deprived myopia (FDM) mouse models were analyzed in a prior study. It was found that lncRNA Gm35369 might be related to myopia and that this lncRNA was mainly located in ganglion cells and horizontal cells [14]. Assessment of the differential expression of lncRNAs in the ocular posterior poles of two guinea pig myopia models (FDM and lens-induced myopia (LIM)) has revealed that lncRNA-related extracellular matrix (ECM), kinase activity, metabolism, ECM receptor interactions, glycosaminoglycan degradation and multiple functional pathways are involved in the pathogenesis of myopia [15].

Research has shown that the retina plays an important role in the pathology of myopia. The nerve fiber layer of the retina in highly myopic ocular hypertension patients is significantly thinned [16]. With the prolongation of FDM, the guinea pig retina becomes thinner, and the ganglion cell numbers and inner and outer nuclear layer thicknesses of the retina decrease [17]. Retinal ganglion cells (RGCs) are cells responsible for transmitting information from the eyes to the brain through the optic nerve [18]. Recent studies have shown that RGCs may be involved in the occurrence and development of myopia. Clinical studies and animal experiments have confirmed that myopia can cause a decrease in the number of RGCs [19, 20]. Early-onset high myopia can cause abnormal axonal projection of RGCs [21]. Stimulating neuropsin in RGCs can prevent experimental myopia in mice [22]. However, the function of lncRNAs in RGCs during the development and progression of myopia is still unknown, and the role of ceRNAs in RGCs in myopia development has not been explored. Therefore, exploring the lncRNA-mediated ceRNA network in RGCs during the development of myopia is of great significance. This will provide a favorable reference value for the exploration and development of gene editing therapeutics for hereditary myopia.

In the present study, we detected the lncRNA and mRNA expression profiles in RGCs between FDM and normal control guinea pigs by RNA sequencing. Next, we constructed the related ceRNA networks based on differentially expressed lncRNAs and mRNAs as well as miRNAs from the miRanda database. We explored potentially important genes and related signaling pathways in myopia with FDM guinea pigs, FDM mice, and high myopia patients. Furthermore, the expression of the lncRNAs/miRNAs/mRNAs was validated by qRT–PCR and related molecular experiments. This study aimed to provide potential therapeutic targets for the occurrence and development of myopia through analysis of the lncRNA‒miRNA–mRNA network in RGCs.

## Materials and methods

### Animals

The experiments adhered to the Association for Research in Vision and Ophthalmology (ARVO) Statement for the Use of Animals in Research. Male colored guinea pigs (2 weeks old) were purchased from Danyang Changyi Experimental Animal Breeding Co., Ltd. (Danyang, China; License no: SYXK (Shanghai) 2018-0019) and used for FDM models. The experimental procedure was approved by the Ethics Committee of Yueyang Clinical Medicine School, Shanghai University of Traditional Chinese Medicine.

Male C57BL/6 mice (3 weeks old) were purchased from Shanghai Bikai Keyi Biotechnology Co., Ltd. (Shanghai, China; License no: SYXK (Shanghai) 2018-0006) and used for FDM models. The experimental procedures in this study were approved by the Animal Care and Ethics Committee at the EYE & ENT Hospital of Fudan University. The animals were housed in an indoor environment with a 12 h day/12 h night cycle, a temperature of 22–24 °C, a relative humidity of 40–60%, and a luminance of approximately 200 lx. They had free access to food, water, and vegetables.

### Induction of FDM models

Guinea pigs were assigned to two groups: the normal control group (NCG) and the FDM group (FDMG) (n = 21/group). All eyes in the NCG were untreated. The right eyes in the FDM group were occluded with latex balloons as translucent masks for 8 weeks as previously described [23], and the fellow eyes remained unoccluded. Throughout the entire experiment, care was taken to ensure that the right eye remained completely covered but that the animal could blink freely. The left eyes of the FDM group animals were used as contralateral controls.

Mice were also assigned to two groups: the normal control group (NCG) and the FDM group (FDMG) (n = 9/group). Monocular FDM was induced in mice as previously described [24]. Briefly, the right eyes of mice were covered with a white translucent occluder that was glued around the eyes for 4 weeks. A plastic collar was fitted around the mouse’s neck to prevent the mouse from removing the occluder.

### Assessment of refraction and axial length

For guinea pigs, after pupil dilation with 0.5% compound tropicamide solution (Santen Pharmaceutical Co., Ltd. Shiga Plant. China), refraction was measured with a streak retinoscope (YZ24; 66 Vision Tech Co., Ltd. China) in the dark [25]. The average refractive index of the horizontal and vertical meridians was taken as the final spherical equivalent (SE). Each eye was measured three times and averaged. Measurements of axial length (AL) were performed by A-scan ultrasonography (KN-1800; Kangning, Jiangsu Province, China) [26]. A drop of 0.4% oxytetracaine hydrochloride (Benoxil; Santen, Osaka, Japan) was applied to each eye, after which ultrasound measurements were made. Ten repeated measurements were taken to calculate the mean.

For mice, ocular refraction was measured in the dark with an eccentric infrared photorefractor developed by Frank Schaeffel [27]. Mice were intraperitoneally anesthetized. A drop of 0.5% compound tropicamide solution was added to each eye; the pupil fully dilated within a few minutes. To avoid cataract formation during anesthesia, the mice were subjected to refractive measurements immediately. Data were recorded automatically by a program designed by Schaeffel. The refraction of each eye was assessed as the mean of 10 sets of measurements. AL was measured using a swept-source optical coherence tomography (OCT) device according to our team's previous study [28]. CASIA2 was used to acquire images. The optical axis of incident light needed to pass perpendicularly through the apex of the cornea during the scan. We confirmed alignment by making adjustments to the mouse position to ensure that the mouse iris was horizontal to the reference line on both the vertical and horizontal image monitoring screens (Fig. 6B). CASIA2 scanning lasted approximately 2.4 s (50,000 A-scans per second). Each measurement was repeated three times.

### Hematoxylin and eosin (H&E) staining and TUNEL staining

H&E staining was performed to observe the eye morphology of guinea pigs, mice, and humans. Eyeballs were fixed in 4% paraformaldehyde and embedded in paraffin. The prepared eye sections were stained with H&E. The eye sections were sealed and observed using a microscope (Olympus Corporation, Japan). The number of RGCs was record in the same area using H&E section. Three different views were taken of each tissue, and the number of cells was averaged.

For the TUNEL assay, eye slices were deparaffinized and rehydrated, and they were then washed with PBS 3 times for 5 min each time. The slices were permeabilized with 0.1% Triton X-100 for 15 min at room temperature and then incubated with a TUNEL Kit (TDT enzyme, dUTP and buffer mixed at a ratio of 1:5:50) at 37 °C in the dark for 2 h. The nuclei were stained with DAPI. Images were collected using a fluorescence microscope (Olympus Corporation, Japan).

### Isolation and culture of RGCs

**Fig. 1:**
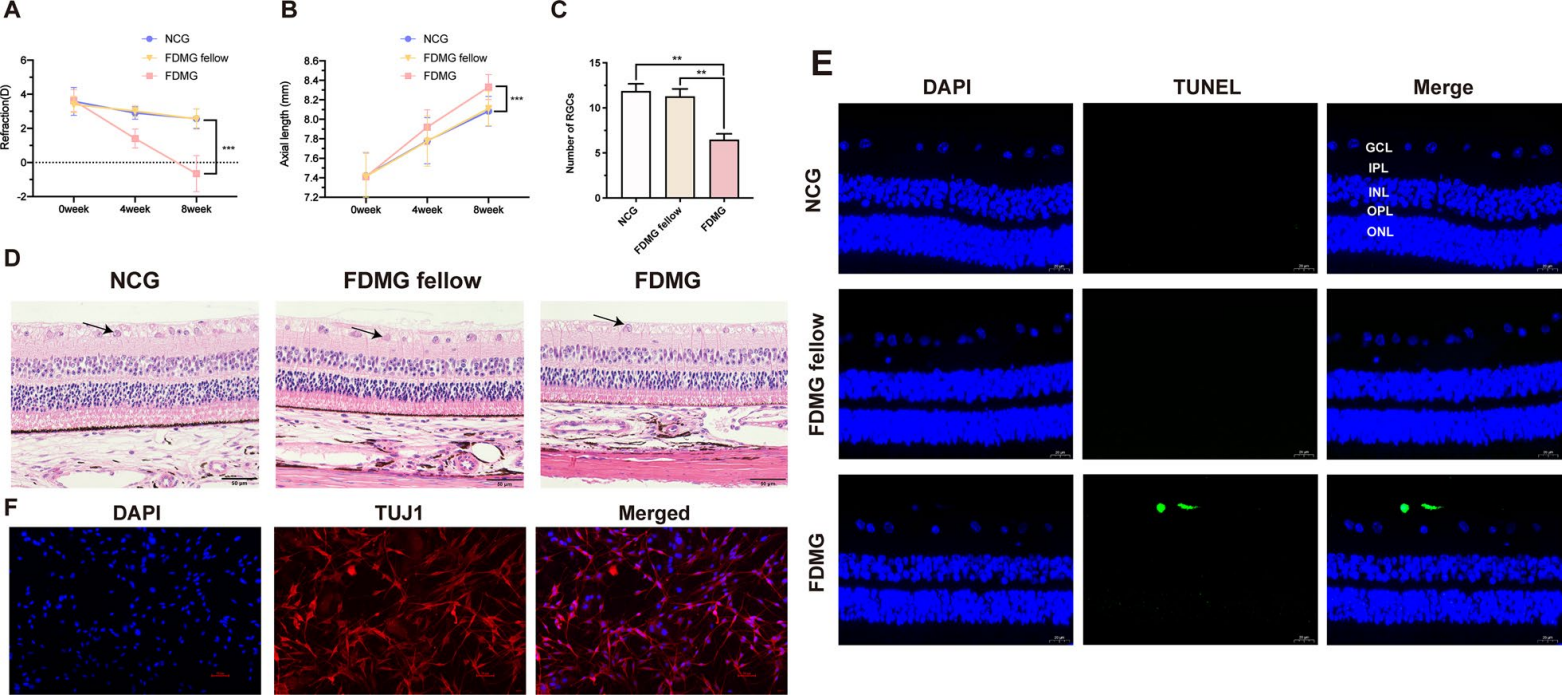
Assessment of FDM model in guinea pigs. **A** Refraction in guinea pigs at three time points (n = 21). **B** Axial length (AL) in guinea pigs at three time points (n = 21). **C** The number of RGCs with H&E-stained retinas in guinea pigs. **D** Morphological observation with H&E-stained retinas in guinea pigs, and arrows indicate RGCs sites (scale bar: 50 µm) (n = 5). **E** TUNEL sections of retinas in guinea pigs. DAPI (blue); apoptotic cell (green) (scale bar: 20 µm) (n = 3). **F** Identification of primary RGCs. DAPI (blue); TUJ1 (red) (scale bar: 50 μm). GCL: ganglion cell layer; IPL: inner plexiform layer; INL: inner nuclear layer; OPL: out plexiform layer; ONL: out nuclear layer. ^***^P < 0.001. NCG: normal control group; FDMG fellow: form-deprived myopia fellow control group; FDMG: form-deprived myopia group

RGCs were isolated from guinea pigs according to the methods in previous studies [29, 30]. The retina was detached from the guinea pig eye cups and trimmed into small pieces in Hank’s balanced salt solution (HBSS, Invitrogen) on ice. Then, the retinal pieces were incubated in papain (Sigma), crude collagenase (Sigma), L-cysteine and 0.02% BSA (Sigma) for 30 min at 37 °C with gentle agitation. After digestion, the retinal tissue was transferred to OVO-1 solution and Ovomucoid (Sigma) for grinding of the cells. The cells were passed through a mesh filter (20 µm pore size) to obtain single cells. The filtered single cells were transferred to a pretreated culture dish containing goat anti-rabbit IgG (HL) antibodies and coated overnight. After the end of negative selection, the cells were transferred again to a culture dish containing goat anti-mouse IgG (HL) antibody. After the end of positive selection, RGCs were adsorbed on the bottom of the culture dish, and an appropriate amount of iCell primary neuronal cell culture system (iCell Bioscience Inc.) was added. Finally, RGCs were inoculated into a 6-well cell culture plate coated with poly-L-lysine and mouse laminin. The purity of the RGCs was determined by immunofluorescence (Fig. 1F).

A TUJ1 antibody (Beyotime, Shanghai, China) was used for RGC immunofluorescence identification. Cells were cultured for 24 h before fixation, washed with PBS, and fixed with 4% PFA. Next, the cells were permeabilized in 0.25% Triton X-100 for 5 min and incubated with 5% BSA. After incubation with a primary antibody against TUJ1 at 4 °C overnight, Alexa Fluor 594-labeled secondary antibody (Proteintech, USA) was added, and the cells were incubated for 2 h at room temperature. DAPI (Solarbio, Beijing, China) was used for nuclear staining for 5 min. All images were obtained with a fluorescence microscope (Olympus, Japan).

### RNA isolation and construction of an RNA library

According to the previous method, high-throughput sequencing was performed on guinea pig RGCs (Oebiotech Company, Shanghai, China) to detect lncRNA and mRNA expression profiles [31]. There were 6 samples (3 FDMG and 3 NCG). Total RNA was extracted using a mirVana™ miRNA Isolation Kit (Thermo). Subsequently, the integrity of RNA was examined with a gel (1%) imaging system (Tanon 2500, Biotanon Co., Ltd), and the concentration was determined with a NanoDrop-2000 (Thermo). LncRNA and mRNA libraries were constructed using TruSeq stranded total RNA with a Ribo-Zero Gold Prep Kit (Illumina). After removal of rRNA, RNA was purified by magnetic beads. Immediately afterward, first- and second-strand cDNAs were synthesized, and library fragments were purified by AMPure XP beads to select cDNA fragments. The sequencing data were deposited in a public repository (Sequence Read Archive, https://www.ncbi.nlm.nih.gov/sra/PRJNA1021213).

### Bioinformatic analysis of mRNA and lncRNA

Clean reads were aligned to the reference genome of *Cavia porcellus* using HISAT2 [32]. The fragments in each gene were counted by StringTie software. Transcripts with coding potential were selected by CNCI, CPC, PLEK and Pfam to obtain predicted lncRNA sequences. Sequencing reads from each sample were aligned to sequences of known lncRNAs, mRNA transcripts and predicted lncRNA sequences by Bowtie2. Fragments per kilobase of exon model per million reads mapped (FPKM) values and counts were obtained from gene quantitative analysis using eXpress. The analysis of differentially expressed (DE) lncRNAs and DE mRNAs was performed by the R package edgeR. Differentially expressed transcripts with *p* values < 0.05 and |log2(FC)| values > 1 were selected. Gene Ontology (GO) and Kyoto Encyclopedia of Genes and Genomes (KEGG) pathway analyses were performed via hypergeometric distribution test.

### LncRNA–miRNA‒mRNA network construction

By supplementing our data with miRNAs in the miRanda database, regulatory relationships between lncRNAs and miRNAs and between miRNAs and mRNAs were predicted. The scores between pairs of ceRNA relationships were calculated in combination with the ceRNA MuTATE method [33], while the probability of sharing some miRNAs among pairs of ceRNA relationships was calculated in combination with the hypergeometric distribution algorithm. The ceRNA relation pairs with high reliability were finally obtained. The ceRNA networks were constructed using Cytoscape (version 3.6.1; http://cytoscape.org/).

### Donor ocular tissues

Normal adult and highly myopic adult eyes (35 to 65 years of age) were obtained from Bright Eye Hospital. This study was approved by the Institutional Review Board of Fudan University and followed the Declaration of Helsinki guidelines. The suitability of the eye tissue was judged by the history of the donors. Donors with syndromic connective tissue diseases (corneal opacities, congenital cataracts, metabolic cataracts, etc.) were excluded. Eyes with late-stage eye disease, including age-related macular degeneration and glaucoma, were excluded. Donors with serious systemic diseases, such as cancer and diabetes, were also excluded. Within 5 h after death, eye cups were collected, and the retina, choroid, and sclera were separated and stored in a − 80 °C refrigerator. All eye tissues were from people of the Asian race, and the sex distribution was balanced.

### Quantitative real-time PCR (qRT‒PCR)

LncRNAs, miRNAs and mRNAs were selected for validation. Total RNA was extracted from guinea pig RGCs, mouse retinas and human retinas with TRIzol reagent (Invitrogen, Carlsbad, CA, USA). For lncRNA and mRNA expression, total RNA was used for cDNA synthesis using a PrimeScript RT Reagent Kit with gDNA Eraser (Takara, Japan). For miRNA expression, cDNA synthesis was performed using an EZ-press microRNA Reverse Transcription Kit (EZBioscience, USA). The resultant cDNA was amplified in a Roche Light Cycler 480 II using TB Green™ Premix Ex Taq™ (Tli RNaseH Plus) (Takara, Japan). The relative amounts of transcripts were analyzed using the 2^−ΔΔCt^ method. GAPDH and U6 were used as references for normalization. The primer sequences for guinea pigs, mice and humans are listed in Additional file 1: Tables S1–S3.

### Western blot (WB)

Total proteins were extracted from guinea pig RGCs and sclerae, mouse retinas and sclerae and human retinas and sclerae. An SDS‒PAGE gel was used to separate equal amounts of proteins, which were transferred to PVDF membranes (Millipore, Boston, MA, USA). The PVDF membranes were blocked with 5% BSA. Subsequently, primary antibodies against c-GMP (Santa Cruz, MA, USA), PKG-1 (CST, MA, USA), MMP-2 (Abcam, Cambridge, UK), HIF-1a (Abcam, Cambridge, UK), t-ERK (Abcam, Cambridge, UK), p-ERK (Abcam, Cambridge, UK), and ACTIN (Beyotime, Shanghai, China) were incubated with the PVDF membranes at 4 °C overnight. The next day, the membranes were incubated with secondary antibodies (1:1000; Beyotime, Shanghai, China). Proteins were detected with an enhanced chemiluminescence kit (Beyotime, Shanghai, China). The gray values of the proteins were analyzed using ImageJ software.

### Immunofluorescence

Dewaxing and antigen repair of paraffin eye sections were performed. Subsequently, the sections were sealed with goat serum for 30 min. Primary antibodies against ADCY1 (Santa Cruz, MA, USA), c-GMP (Santa Cruz, MA, USA) and APELIN (Servicebio, Wuhan, China) were added to the sections, and the slices were placed flat in a wet box at 4 °C for overnight incubation. The next day, the sections were incubated with secondary antibodies at room temperature in the dark for 50 min and then incubated with DAPI staining solution at room temperature in the dark for 10 min. Anti-fluorescence quenching sealing agent was applied to the sections. All images were obtained using a fluorescence microscope. The integral optical density (IOD) intensity was measured by ImageJ software.

### Immunohistochemistry

Eye sections were heated at 60 °C and deparaffinized in xylene and gradient alcohol solutions. Then, the sections were placed in a repair box filled with citric acid antigen repair solution (pH 6.0) for antigen retrieval and incubated in a 3% hydrogen peroxide solution at room temperature in the dark for 25 min. The sections were blocked with 3% BSA for 30 min and then stained with an anti-ADCY1 antibody (Santa Cruz, MA, USA) and anti-cGMP antibody (Santa Cruz, MA, USA) overnight at 4 °C. The next day, the sections were incubated with secondary antibody for 50 min, and color development was performed with DAB solution. Finally, the sections were dyed with hematoxylin for 3 min and observed with a light microscope. The IOD intensity was measured with ImageJ software.

### Statistical analysis

SPSS 25.0 statistical software (IBM, Armonk, NY, USA) was used for statistical analyses. Experiments were repeated at least three times. All data in the different experimental groups are presented as the mean ± SE and were analyzed using unpaired Student’s t tests, one-way ANOVA and the Bonferroni t test. *P* < 0.05 was considered to indicate statistical significance.

## Results

### Establishment of FDM guinea pigs and FDM mice

Visual form deprivation in guinea pigs was started at approximately 2 weeks (n = 21). There was no significant difference in refraction among the groups before form deprivation (Fig. 1A) (*P* > 0.05). After 8 weeks of form deprivation, the refraction (in diopters, D) in the FDMG (− 0.65 ± 1.05 D) was shifted toward myopia compared to that in the NCG (+ 2.57 ± 0.57 D) and FDMG fellow (+ 2.54 ± 0.60 D) (*P* < 0.001) (Fig. 1A). After form deprivation for 8 weeks, compared with the NCG (8.08 ± 0.15 mm) and FDMG fellow (8.11 ± 0.17 mm), the axial length of the eye was increased in the FDMG (8.33 ± 0.12 mm) (*P* < 0.001) (Fig. 1B).

Upon establishment of FDM mice, the refraction in the FDMG (− 4.01 ± 4.39 D) was more myopic than that in the NCG (+ 9.19 ± 0.61 D) and FDMG fellow (+ 8.27 ± 1.88 D) (*P* < 0.001) (Fig. 6A), and increased axial elongation was observed in the FDMG (3.47 ± 0.03 mm) compared with the NCG (3.29 ± 0.08 mm) and FDMG fellow (3.36 ± 0.09 mm) (*p* < 0.05) (Fig. 6C). These results indicated that FDM guinea pig and mouse models were established successfully.

### Observation of retinas and detection of RGC apoptosis

We confirmed the retinal structure of FDM guinea pig eyes with H&E staining (Fig. 1D). The retinal structure in the NCG and FDMG fellow was clear, the thickness of the ganglion cell layer and the number of ganglion cells were normal, and the nucleoli of the nucleus were clearly arranged. After 8 weeks of form deprivation, the retina of FDMG was thinner, the number of RGCs in FDMG was reduced than that in NCG and FDMG fellow (*p* < 0.01) (Fig. 1C), the distribution of RGCs was uneven, and the structure of the other layers was clear. The H&E results showed that the retinas of FDM mice became thinner and that the distribution of RGCs was sparse, which is consistent with the situation in FDM guinea pigs (Fig. 6D).

We further analyzed the apoptosis of RGCs in the retina by TUNEL immunohistological assay (Fig. 1E). Compared with that in the NCG and FDMG fellow, the TUNEL-positive signal in the RGCs of FDM guinea pigs increased. These results showed that the model of FDM guinea pigs had been successfully established and that the number of RGCs was decreased and that RGC apoptosis was increased in FDM guinea pigs.

### Analysis of lncRNA expression profiles in RGCs between FDM and normal control guinea pigs

**Fig. 2:**
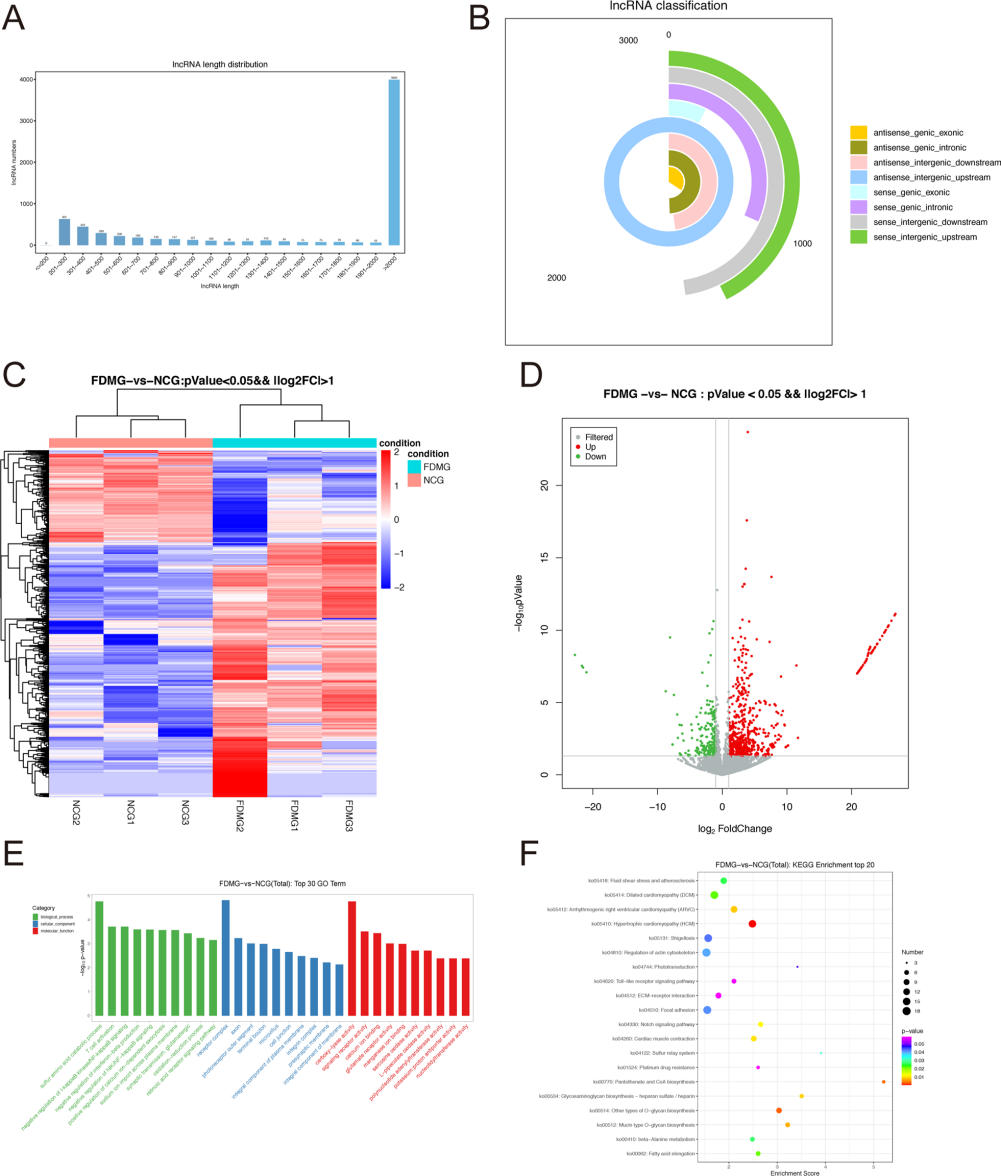
The DE lncRNAs were assessed by the RNA sequencing data in FDMG and NCG (three for FDMG and three for NCG). **A** LncRNAs length distribution. **B** The distribution pie chart of lncRNA types. **C** Volcano plot and **D** heatmap of DE lncRNAs between FDMG and NCG (log2FC > 1 and *P* < 0.05). **E** The top 30 GO enrichment analysis of the genes between FDMG and NCG. **F** Top 20 KEGG enrichment analysis of the genes between FDMG and NCG. DE: differentially expressed; NCG: normal control group; FDMG: form-deprived myopia group

To explore the function of lncRNAs in RGCs during the development and progression of myopia, we extracted RGCs from guinea pig retinas. Primary cells extracted from guinea pig retinas were identified as RGCs by immunofluorescence staining with the RGC-specific marker TUJ1 (red) (Fig. 1F). By screening differentially expressed lncRNAs in RGCs from NCG and FDMG guinea pigs by RNA sequencing, we found that the diverse length distribution of lncRNAs was different and that the length of most lncRNAs was less than 2000 nucleotides (Fig. 2A). The proportions of different types of lncRNAs, such as antisense-genic, antisense-intergenic, sense-genic and sense-intergenic lncRNAs, are illustrated (Fig. 2B).

As shown in Fig. 2C, D, hierarchical clustering analysis showed that there were 873 differentially expressed (DE) lncRNAs (log2FC > 1, *P* < 0.05) between the FDMG and NCG, of which 642 were upregulated and 232 were downregulated. Additional file 1: Table S4 presents the top 10 differentially upregulated and downregulated lncRNAs between FDMG and NCG.

To further investigate the response of the lncRNAs, the parental genes of the DE lncRNAs were used in GO clustering and KEGG pathway enrichment (Fig. 2E, F). GO enrichment analysis showed that DE lncRNAs in the FDMG were enriched in biological processes such as sulfur amino acid catabolic process and T-cell activation; cellular components such as the receptor complex, axon and photoreceptor outer segment; and molecular functions such as carboxy-lyase activity, signaling receptor activity and calcium ion binding (Fig. 2E). The KEGG pathways were mainly related to the Toll-like receptor signaling pathway, Notch signaling pathway, ECM-receptor interaction and Mucin type O-glycan biosynthesis (Fig. 2F). Together, these results suggest that lncRNAs in RGCs play a critical role in myopia pathogenesis.

### Analysis of mRNA expression profiles in RGCs between FDM and normal control guinea pigs

**Fig. 3:**
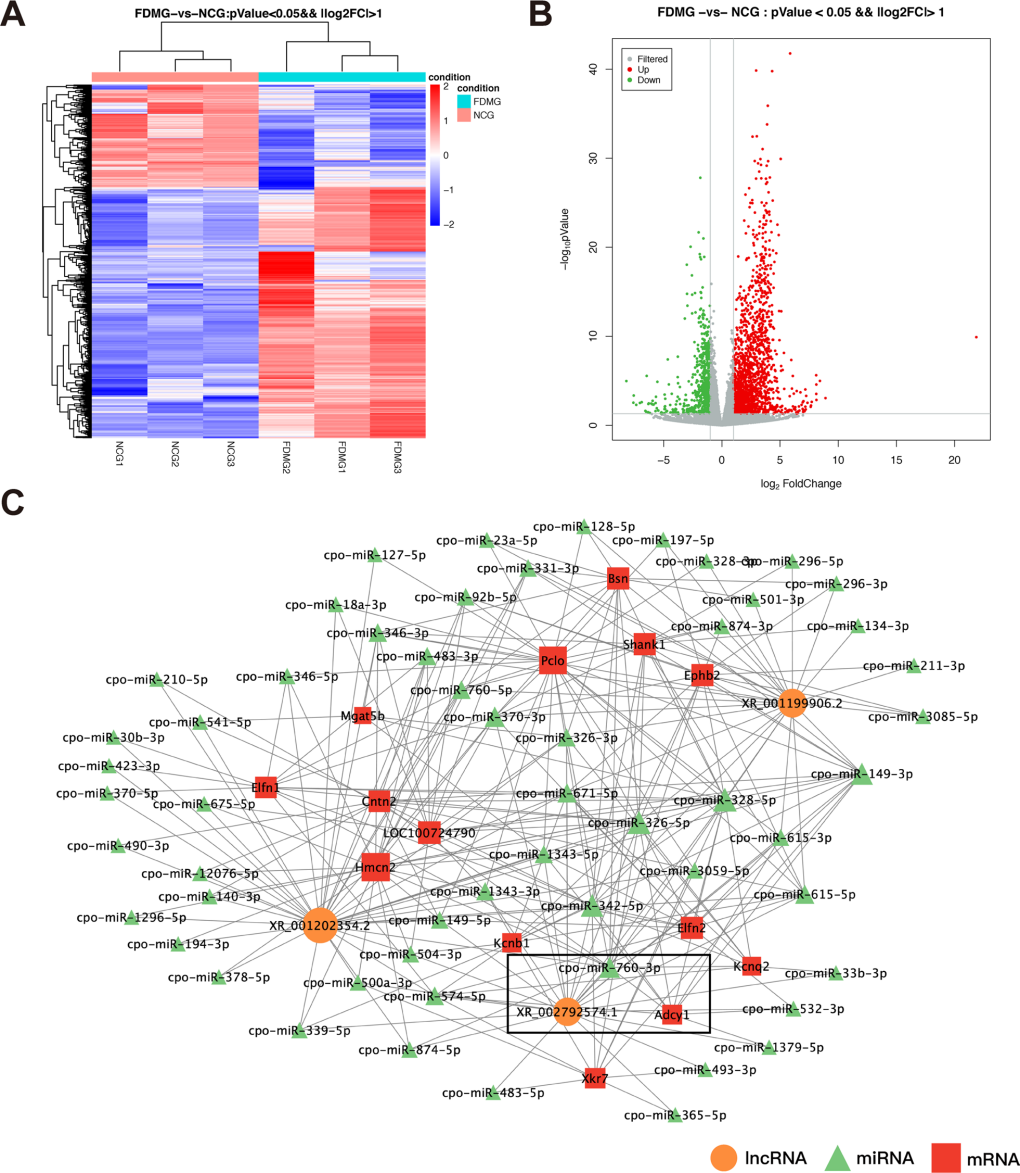
The DE mRNAs were analyzed by the RNA sequencing data in FDMG and NCG (three for FDMG and three for NCG) and lncRNA-miRNA-mRNA network analysis. **A** Volcano plot and **B** heatmap of DE mRNAs between FDMG and NCG (log2FC > 1 and *P* < 0.05). **C** LncRNA-miRNA-mRNA network analysis between FDMG and NCG. Orange, green and red represent lncRNAs, miRNAs and mRNAs, respectively. DE: differentially expressed; NCG: normal control group; FDMG: form-deprived myopia group

We further explored DE mRNAs in RGCs from three normal control and three FDM guinea pigs by RNA sequencing. As shown in Fig. 3A, B, there were 2480 DE mRNAs (log2FC > 1, *P* < 0.05), including 1761 upregulated and 719 downregulated mRNAs, in the FDMG compared to the NCG. The top 10 differentially up- and downregulated mRNAs are presented in Additional file 1: Table S5. In addition, we performed GO enrichment and KEGG pathway analyses to identify biological processes or potential pathways related to FDM in guinea pigs (Additional file 1: Fig. S1).

### LncRNA–mRNA network construction and lncRNA–miRNA–mRNA network construction

To elucidate the potential interactions of DE lncRNAs and mRNAs, we screened the coexpressed genes in the FDMG and NCG and constructed a lncRNA‒mRNA coexpression network. According to the role of mRNA‒lncRNA interaction in the ceRNA relationship, 171,943 pairs of mRNA‒lncRNA relationships with positive correlations were identified. The top 100 lncRNA‒mRNA pairs with the largest MuTATE score in the ceRNA analysis results were plotted (Additional file 1: Fig. S2). Importantly, lncRNA-XR_001202354.2, lncRNA-XR_001199906.2 and lncRNA-XR_002792574.1 were connected by a large number of mRNAs.

Next, we constructed the related ceRNA networks based on DE lncRNAs and mRNAs as well as miRNAs from the miRanda database. The Miranda v3.3a program was used to predict the miRNA‒lncRNA and miRNA‒mRNA regulatory pairs. We identified 3060 miRNA‒lncRNA regulatory pairs and 15,846 miRNA‒mRNA regulatory pairs. Finally, we constructed a ceRNA network based on 200 mRNA‒miRNA–lncRNA regulatory triads among the top 100 mRNA‒lncRNA pairs in the ranking results of the ceRNA analysis (Fig. 3C). These results further demonstrate that lncRNA-XR_001202354.2, lncRNA-XR_001199906.2 and lncRNA-XR_002792574.1 might be involved in the mechanisms of myopia.

### Functional analysis of the ceRNA network

**Fig. 4:**
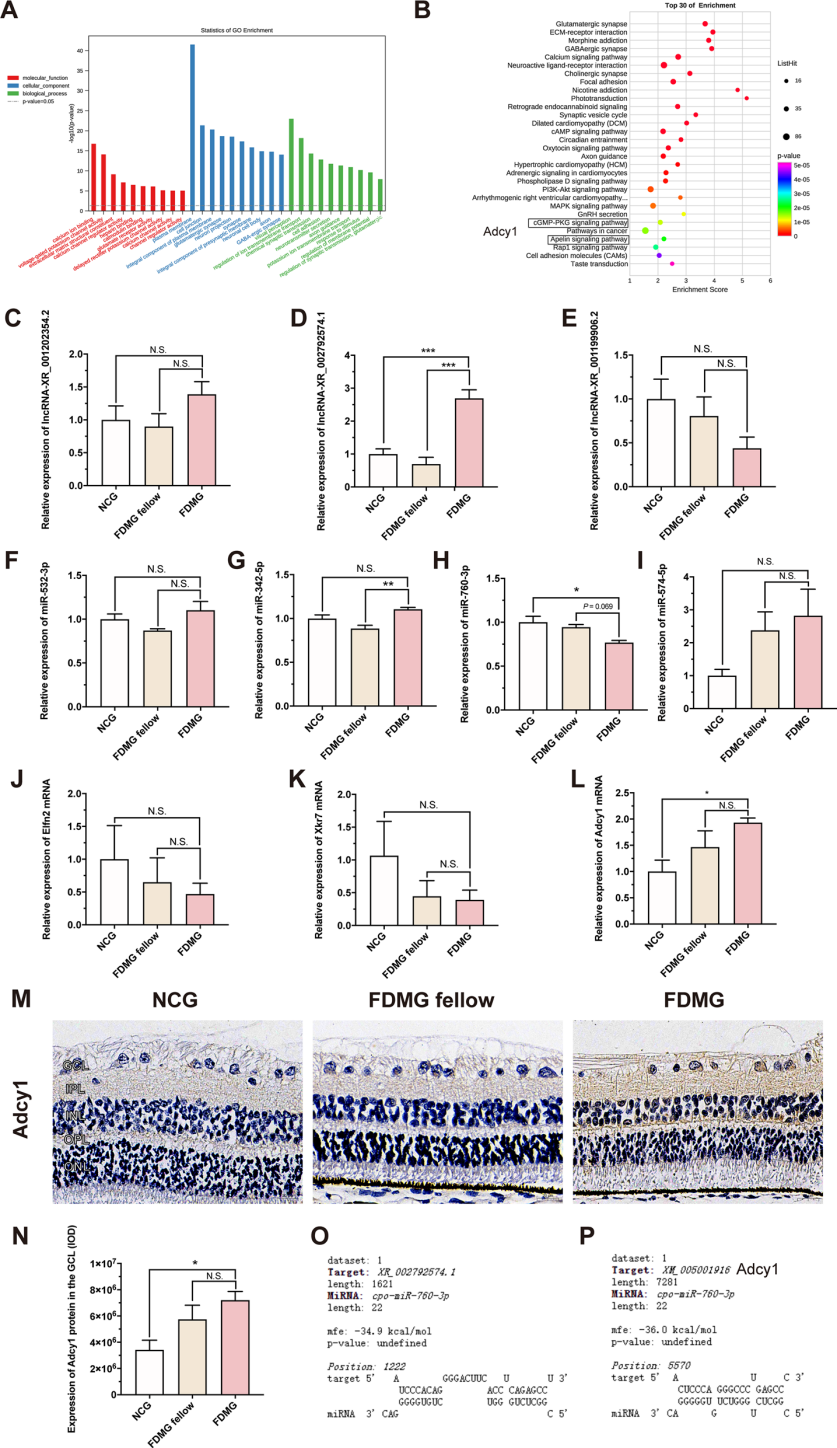
Functional analysis of ceRNA network and validations of differential RNA expression of ceRNA network in guinea pigs. **A** Top 10 enriched GO terms of enriched mRNA target genes in ceRNA networks. **B** Top 30 KEGG enrichment analysis of enriched mRNA target genes in ceRNA networks. **C**–**E** The key lncRNA expressions of lncRNA-XR_001202354.2, lncRNA-XR_002792574.1 and lncRNA-XR_001199906.2 were measured by qRT-PCR. **F**–**I** The relative expressions of miR-532-3p, miR-342-5p, miR-760-3p and miR-574-5p were measured by qRT-PCR. **J**–**L** The relative mRNA expressions of Elfn2, Xkr7 and Adcy1 were measured by qRT-PCR. **M** Images of Adcy1 (brown) immunohistochemistry in retina from guinea pigs (scale bar: 20 µm). **N** IOD analysis of Adcy1 expression in GCL from guinea pig. **O** The targeting relationship between lncRNA-XR_002792574.1 and miR-760-3p was predicted. **P** The targeting relationship between miR-760-3p and Adcy1 was predicted. For each group, n = 4–6. ^*^P < 0.05, ^**^P < 0.01, ^***^P < 0.001. NCG: normal control group; FDMG fellow: form-deprived myopia fellow control group; FDMG: form-deprived myopia group

To further investigate the biological functions of lncRNAs in lncRNA-associated ceRNA networks, GO and KEGG analyses were performed for target genes directly interacting with lncRNAs (Fig. 4A, B). The GO analysis showed that the terms were enriched in the molecular functions of calcium ion binding and calcium channel regulator activity; the cellular components of neuron projection, neuronal cell body and GABAergic synapse; and the biological processes of visual perception and chemical synaptic transmission and neurotransmitter secretion (Fig. 4A). KEGG enrichment analysis predicted that the main pathways included the GABAergic synapse pathway, the calcium signaling pathway, the MAPK signaling pathway, the cAMP signaling pathway, the cGMP­PKG signaling pathway and the apelin signaling pathway (Fig. 4B).

### Validation of differential RNA expression of the ceRNA network

Based on the ceRNA results, we validated the important lncRNAs, miRNAs and mRNAs by qRT‒PCR. Three DE lncRNAs with the highest ceRNA scores were selected. The results demonstrated that the expression of lncRNA-XR_002792574.1 was significantly upregulated in guinea pig RGCs of the FDMG compared to the NCG and FDMG fellow (*p* < 0.001) (Fig. 4D), but the expression of lncRNA-XR_001202354.2 and lncRNA-XR_001199906.2 showed no significant difference in guinea pig RGCs (*P* > 0.05) (Fig. 4C, E). Moreover, we also demonstrated that the expression of lncRNA-XR_377380.2 increased in the retina of FDM mice with homology with guinea pig lncRNA-XR_002792574.1 compared with that in the NCG and FDMG fellow (*p* < 0.05) (Fig. 6F).

We further detected the expressions of the first three mRNAs closely related to lncRNA-XR_002792574.1 in the ceRNA analysis. Among the three mRNAs, Adcy1 expression in guinea pig RGCs was consistent with the RNA sequencing data analysis results (*p* < 0.05) (Fig. 4J–L). Immunohistochemistry confirmed that the expression of Adcy1 in the GCL of FDM guinea pigs was increased compared with that in the GCL of NCG guinea pigs (Fig. 4M, N) (*P* < 0.05). The result was in accordance with previous PCR data.

Finally, we verified the first four miRNAs that may interact with both lncRNA-XR_002792574.1 and Adcy1 mRNA in the ceRNA analysis (Fig. 4F–I). The PCR results showed that compared with that in the NCG, miR-760-3p expression was downregulated in FDM guinea pig RGCs (*p* < 0.05) (Fig. 4H). As expected, the PCR results showed that the expression of miR-760-3p and Adcy1 mRNA in the retinas of FDM mice was consistent with that in the retinas of FDM guinea pigs (*p* < 0.01) (Fig. 6G, H). Immunofluorescence results also further confirmed that the expression of Adcy1 in the RCGs of FDM mice was higher than that in the RCGs of NCG and FDMG fellow mice (*p* < 0.01) (Fig. 6I, J). We further used the Bibiserv database to predict the targeting relationships between lncRNAs and miRNAs and between miRNAs and mRNAs. The results showed that lncRNA-XR_002792574.1 can target miR-760-3p and that miR-760-3p can target Adcy1 (Fig. 4O, P). We further used the Bibiserv database to predict the targeting relationship. The results also showed that miR-760-3p can target Adcy1 in mice (Fig. 6E). Altogether, these results indicate that lncRNA-XR_002792574.1 may be involved in the development of myopia through the miR-760-3p/Adcy1 pathway in RGCs (Fig. 3C).

### Analysis of potential related regulatory cGMP/PKG and apelin signaling pathways of lncRNA-XR_002792574.1 in FDM guinea pigs and FDM mice

**Fig. 5:**
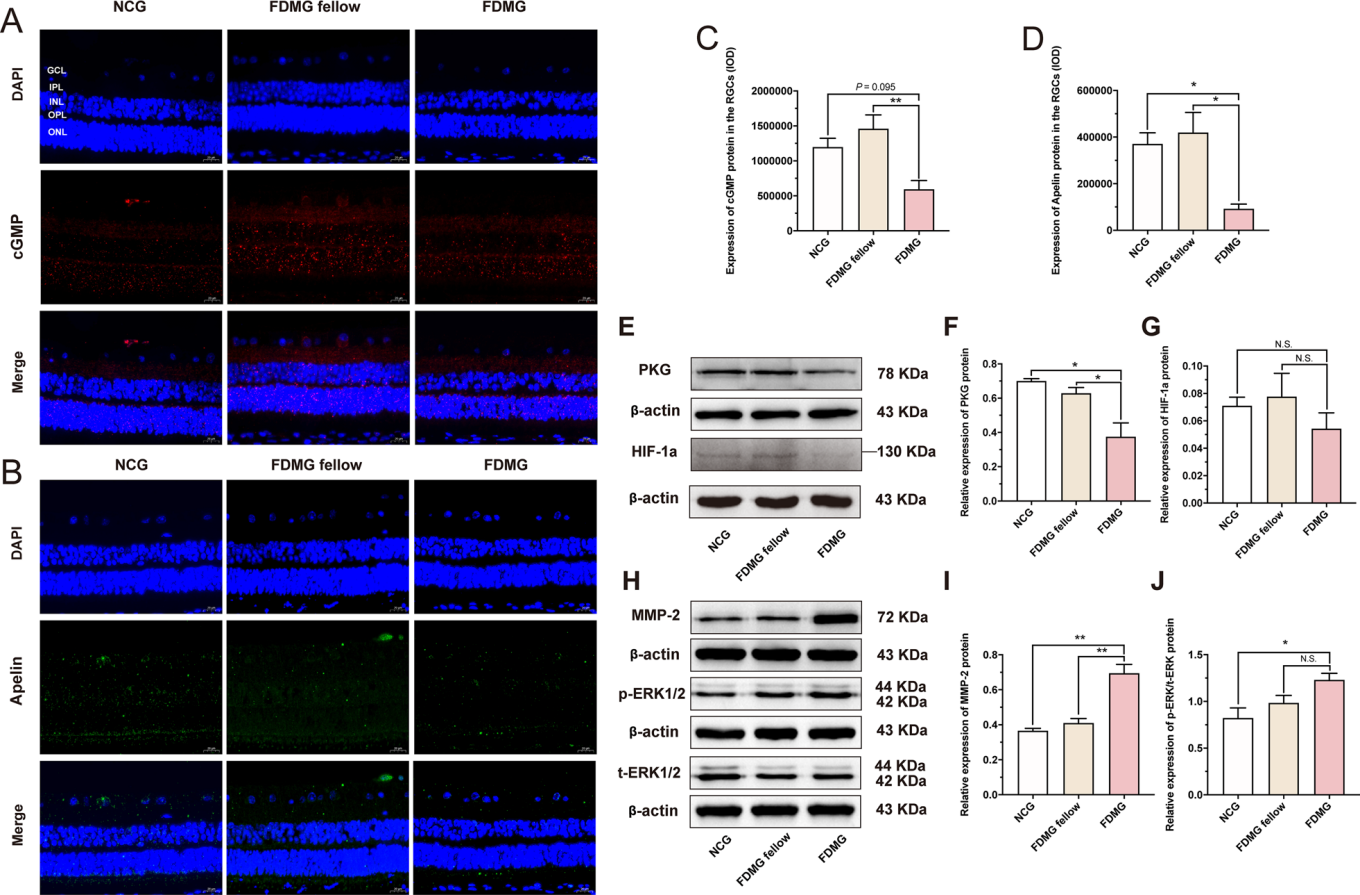
Analysis of related regulatory cGMP/PKG and apelin pathways of lncRNA-XR_002792574.1/miR-760-3p/Adcy1 axis in FDM guinea pig RGCs and its potential relationship with sclera remodeling. **A** Images of cGMP (red) immunofluorescence in retina from guinea pigs with nucleus (blue) (scale bar: 20 µm). **B** Images of Apelin (green) immunofluorescence in retina from guinea pigs with nucleus (blue) (scale bar: 20 µm). **C**, **D** IOD analysis of cGMP expression (**C**) and Apelin (**D**) in RGCs from guinea pig. **E**–**G** Western blot analysis of PKG protein expression (**E**, **F**) and HIF-1a protein expression (**E**, **G**) in guinea pig RGCs. **H**–**J** Western blot analysis of MMP-2 protein expression (**H**, **I**) and p-ERK/t-ERK protein expression (**H**, **J**) in sclera from guinea pigs. For each group, n = 3–6. ^*^P < 0.05, ^**^P < 0.01, ^***^P < 0.001. NCG: normal control group; FDMG fellow: form-deprived myopia fellow control group; FDMG: form-deprived myopia group

To further analyze the function of lncRNA-XR_002792574.1 in FDM guinea pigs and FDM mice, we analyzed the potential regulatory pathways of lncRNA-XR_002792574.1 in RGCs based on the ceRNA results. We noted that the Adcy1 mRNA downstream of lncRNA-XR_002792574.1 was closely related to the cGMP/PKG and apelin pathways according to KEGG enrichment analysis in the ceRNA network (Fig. 4B). Therefore, we examined the cGMP/PKG and apelin pathways in RGCs. Immunofluorescence results showed that cGMP expression in FDM guinea pig RGCs was significantly decreased compared with that in the FDMG fellow (*p* < 0.01) (Fig. 5A, C). Moreover, Western blot analysis showed that PKG expression in FDM guinea pig RGCs was also apparently lower than that in NCG and FDMG fellow (*p* < 0.05) (Fig. 5E, F). Regarding apelin expression, immunofluorescence results showed that there was lower apelin staining detected in FDM guinea pig RGCs than in the NCG and FDMG fellow (*p* < 0.05) (Fig. 5B, D).

Consistent with the results in FDM guinea pigs, compared with that in the NCG and FDMG fellow mice, the expression of cGMP was downregulated in the RGCs of FDM mice (*p* < 0.05) (Fig. 7A, C), and the expression of PKG was also significantly decreased in the retinas of FDM mice (*p* < 0.01) (Fig. 7F, G). The immunofluorescence results showed that the expression of apelin in the RGCs of FDM mice was lower than that in the RGCs of NCG and FDMG fellow mice (*p* < 0.05) (Fig. 7B, D), and the PCR results further showed that apelin expression was reduced in the retinas of FDM mice compared with the retinas of NCG mice (*p* < 0.01) (Fig. 7E).

These results indicate that the lncRNA-XR_002792574.1/miR-760-3p/Adcy1 axis in FDM guinea pigs and FDM mice might have a potential relationship with the cGMP/PKG and apelin signaling pathways. LncRNA-XR_002792574.1 might inhibit the cGMP/PKG and apelin signaling pathways in RGCs through miR-760-3p/Adcy1, thereby causing RGC damage in FDM guinea pigs and FDM mice.

### Analysis of the potential relationship of lncRNA-XR_002792574.1 with retinal hypoxia and scleral remodeling in FDM guinea pigs and FDM mice

Previous studies have pointed out that local hypoxia plays an important role in the development of myopia [34]. To explore whether the retina is hypoxic in myopia and whether the lncRNA-XR_002792574.1/miR-760-3p/Adcy1 axis in RGCs is related to retinal hypoxia, we detected the expression of HIF-1a in the retina. The results showed that retinal HIF-1α levels in FDM guinea pigs were not significantly different from those in NCG and FDMG guinea pigs (*p* > 0.05) (Fig. 5E, G). Consistent with the results in guinea pigs, Western blotting results showed that the levels of HIF-1a in the retinas of FDM mice did not change (*p* > 0.05) (Fig. 7F, H).

Scleral remodeling plays a vital role in the occurrence and development of myopia [35, 36]. To explore whether the lncRNA-XR_002792574.1/miR-760-3p/Adcy1 axis in FDM guinea pig and FDM mice are related to scleral remodeling, we assessed the ERK-MMP-2 pathway in FDM guinea pig and FDM mouse sclera by Western blotting. Figure 5H–J shows that the MMP-2 and p-ERK/t-ERK protein expression levels in FDM guinea pigs were significantly increased compared with those in NCG guinea pigs (*p* < 0.05).

In FDM mice, WB results showed that MMP-2 expression in the sclerae of FDM mice was significantly increased compared with that in the sclerae of NCG and FDMG mice (*p* < 0.001) (Fig. 7I, J). In addition, compared with the NCG group, p-ERK/t-ERK protein expression in the sclerae of FDM mice tended to increase (*p* = 0.192) (Fig. 7I, K). Altogether, these results indicate that the lncRNA-XR_002792574.1/miR-760-3p/Adcy1 axis in FDM guinea pigs and FDM mice may be associated with the scleral remodeling-related ERK-MMP-2 pathway and that there is a positive correlation between retinal lncRNA-XR_002792574.1 and scleral remodeling in FDM guinea pigs and FDM mice.

### The study of the regulatory cGMP/PKG and apelin signaling pathways related to miR-760-3p/Adcy1 in adult retinas with high myopia

Based on the guinea pig and mouse results, we further collected eye samples from highly myopic adult donors to validate miR-760-3p/Adcy1 and the cGMP/PKG and apelin pathways. First, the H&E results showed that the retinas in highly myopic adults became thinner with a sparse distribution of RGCs, which was consistent with the findings in FDM guinea pigs and mice (Fig. 8K). PCR showed that miR-760-3p expression in retinas was also lower in highly myopic adults than in healthy control adults (*p* < 0.05) (Fig. 8A), and Adcy1 expression was increased in highly myopic adults compared to healthy control adults (*p* = 0.072) (Fig. 8B). In addition, the binding site of human miR-760 to Adcy1 was predicted with the Bibiserv database. The results showed that miR-760 can target Adcy1 (Fig. 8C). Similarly, immunofluorescence confirmed that Adcy1 staining in RGCs of highly myopic adults had an upward trend (Fig. 8M).

Furthermore, we explored the cGMP/PKG and apelin pathways in the retinas of adults with high myopia. Western blot results showed that the cGMP/PKG pathway was inhibited in the retinas of adults with high myopia compared with those of healthy control adults (*p* < 0.01) (Fig. 8D–F). Immunohistochemical results also showed that cGMP staining was decreased in RGCs of adults with high myopia (Fig. 8L). With regard to apelin, the immunofluorescence results showed that apelin staining in the RGCs of highly myopic adults was reduced (Fig. 8N). This result was consistent with the guinea pig and mouse results. In conclusion, these findings indicate that miR-760-3p/Adcy1 may be involved in the development of myopia in highly myopic adults, that the cGMP/PKG pathway in highly myopic adults is inhibited, and that the apelin pathway shows a downward expression trend in highly myopic adults.

### The study of the potential relationship between miR-760-3p/Adcy1 and scleral remodeling in highly myopic adults

Consistent with the results of guinea pigs and mice, HIF-1a levels in the retinas of highly myopic adults also showed no changes (*p* > 0.05) (Fig. 8D, G). We further explored the potential connection between miR-760-3p/Adcy1 and scleral remodeling in the sclerae of adults with high myopia. As shown in Fig. 8H, I, compared to that in healthy control adults, MMP-2 expression in the sclera was heightened in highly myopic adults (*p* = 0.051). Moreover, p-ERK/t-ERK protein expression in the sclerae of highly myopic adults was also increased compared with that in healthy control adults (*p* < 0.05) (Fig. 8H, J). Therefore, through clinical samples of high myopia, we further demonstrated that miR-760-3p/Adcy1 in the retinas of adults with high myopia may be associated with the scleral remodeling-related ERK-MMP-2 pathway.

## Discussion

Recent studies have shown that lncRNAs play a crucial role in the pathogenesis of myopia [15, 37]. It has been found that lncRNA Gm35369 might be related to myopia, and this lncRNA is mainly located in ganglion cells and horizontal cells [14]. Studies have reported that RGC alterations are associated with the occurrence and development of myopia, including changes in the number of RGCs, abnormal axonal projections and changes in protein expression in RGCs [19–22, 38]. However, whether lncRNAs in RGCs are involved in the pathogenesis of myopia and the role of lncRNAs as ceRNAs in myopia are still largely unknown. In the present study, we assessed the differential expression patterns of lncRNAs and mRNAs in RGCs between FDM and normal control guinea pigs. Furthermore, we constructed related ceRNA networks and explored potentially important genes and related signaling pathways in FDM guinea pigs with myopia. In addition, the potentially important genes and related pathways of FDM guinea pigs were consistent with FDM mice and high myopia patients.

**Fig. 6:**
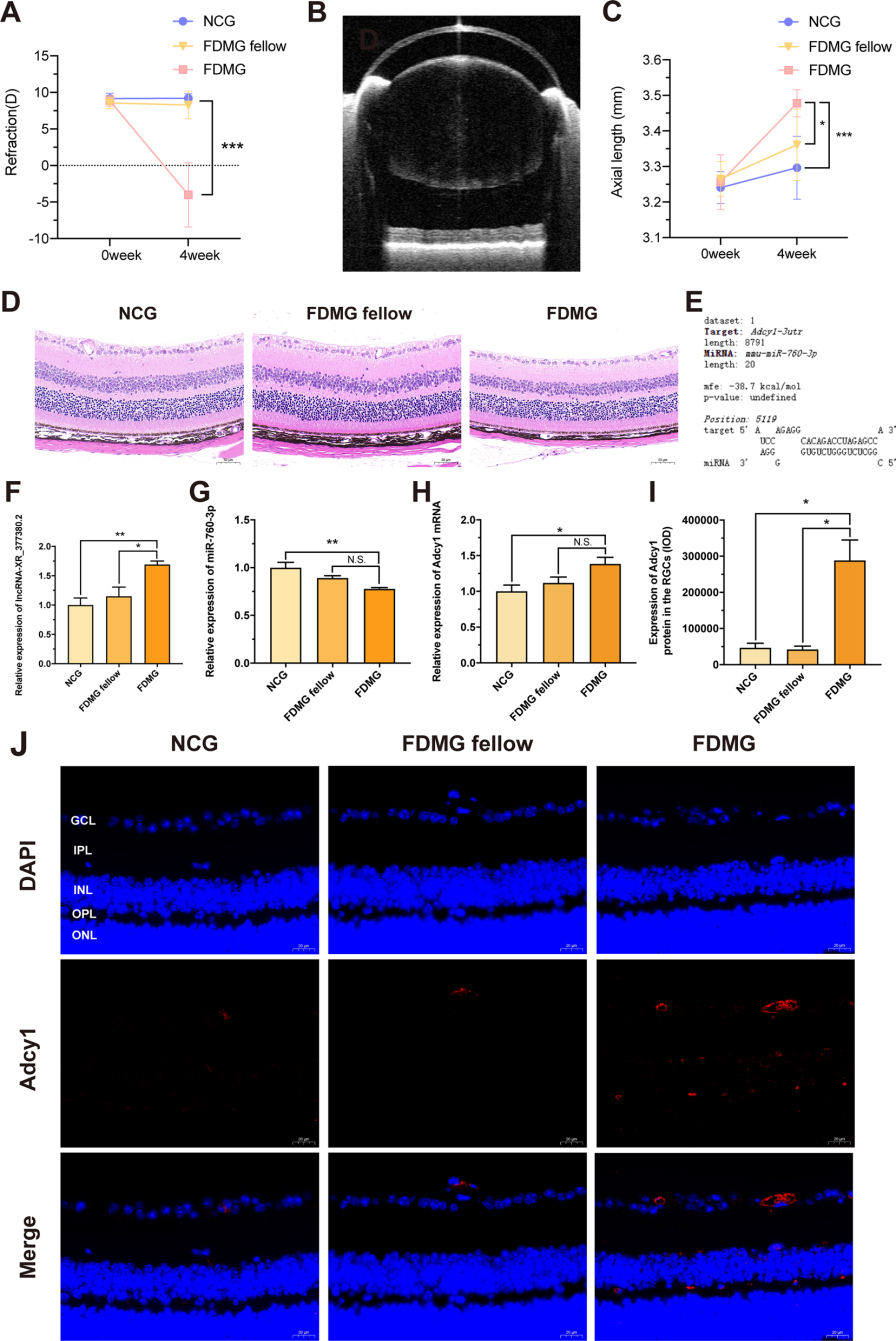
Assessment of FDM model and further validations of miR-760-3p/Adcy1 in mice. **A** Refraction in mice at two time points (n = 9). **B** Representative image of the axial length (AL) in mice. **C** AL in mice at two time points (n = 9). **D** Morphological observation with H&E-stained retinas in mice (scale bar: 50 µm) (n = 5–6). **E** The targeting relationship between miR-760-3p and Adcy1 was predicted. **F** The relative expressions of lncRNA-XR_377380.2 (homologue of guinea pig lncRNA-XR_002792574.1) were measured by qRT-PCR. **G** The relative expressions of miR-760-3p were measured by qRT-PCR. **H** The relative expressions of Adcy1 mRNA were measured by qRT-PCR. **I** IOD analysis of Adcy1 expression in RGCs from mice. **J** Images of Adcy1 (red) immunofluorescence in retina from mice with nucleus (blue) (scale bar: 20 µm). ^*^P < 0.05, ^**^P < 0.01, ^***^P < 0.001. NCG: normal control group; FDMG fellow: form-deprived myopia fellow control group; FDMG: form-deprived myopia group

First, we established FDM guinea pig and FDM mice models, and collected eyeball samples of highly myopic adults. We found that the refraction in FDM guinea pigs and FDM mice was shifted toward myopia, with a 3.19 D difference between FDMD fellow and FDMG eyes in guinea pigs (Fig. 1A) and 12.28 D difference between FDMD fellow and FDMG eyes in mice (Fig. 6A). Similarly, the axial length of the eye was increased in FDM guinea pigs and FDM mice (Figs. 1B, 6C). We further confirmed the retinal structure with H&E staining (Fig. 1C, D). Consistent with previous studies [17], the retinas were thinned, the numbers of RGCs were reduced in the FDM guinea pigs. The H&E also results showed retinal thinning and sparse RGC distribution in both FDM mice and highly myopic adults, consistent with the findings in FDM guinea pigs (Figs. 6D, 8K). The TUNEL results showed that RGC apoptosis was increased in FDM guinea pigs (Fig. 1E).

To explore the lncRNA-mediated ceRNA network in RGCs in myopia, we extracted primary RGCs from guinea pig retinas for RNA sequencing (Fig. 1F). In sequencing analysis of the eye, we found that there were 873 DE lncRNAs between the FDMG and NCG, of which 642 were upregulated and 232 were downregulated lncRNAs (Fig. 2C, D). GO analysis showed that DE lncRNAs in FDM guinea pigs were mostly associated with the photoreceptor outer segment and signaling receptor activity (Fig. 2E). Studies have shown that the decrease in photoreceptor outer segment layer thickness in children and adolescents with unilateral high myopia and amblyopia is related to the decrease in visual acuity [39]. Activation of specific G protein-coupled receptors can regulate a variety of neurological functions. It has been reported that the activation of specific G protein-coupled receptors can inhibit the T-type Ca^2+^ current in rat RGCs, thus protecting RGCs [40].

The KEGG pathways were mainly related to the Notch signaling pathway, ECM-receptor interaction and mucin type O-glycan biosynthesis (Fig. 2F). Previous myopia studies have also revealed KEGG enrichment of ECM–receptor interaction and mucin type O-glycan biosynthesis pathways for differentially expressed lncRNAs in the ocular posterior poles of guinea pigs in the FDM and lens-induced myopia (LIM) models [15]. Studies have shown that the Notch signaling pathway contributes to the overexpression of ECM proteins and promotes retinal fibrosis [41]. Retinal fibrosis in myopic guinea pigs is obvious, and retinal thickness is reduced, which leads to abnormal physiological function of the retina in myopic guinea pigs [42]. Therefore, the Notch signaling pathway may be associated with myopic retinal fibrosis. Together, these results suggest that lncRNAs in RGCs play a critical role in myopia pathogenesis.

We further identified 2480 DE mRNAs in RGCs in FDM guinea pigs compared to normal control guinea pigs, including 1761 upregulated and 719 downregulated mRNAs (Fig. 3A, B). In addition, we performed mRNA-related GO enrichment and KEGG pathway analysis (Additional file 1: Fig. S1). Research has revealed that lncRNAs can bind to miRNAs as ceRNAs to restore the expression and activity of downstream mRNAs [43]. There are regulatory networks among lncRNAs, miRNAs and mRNAs that regulate cell proliferation, apoptosis, migration and invasion [44]. Therefore, we constructed a ceRNA network to explore the function of lncRNAs in FDM guinea pigs (Fig. 3C). Based on the ceRNA results, we validated three DE lncRNAs with the highest ceRNA scores. The results further demonstrated that the expression of lncRNA-XR_002792574.1 significantly upregulated in FDM guinea pig RGCs compared to NCG and FDMG fellow RGCs (Fig. 4D). Moreover, the expression of lncRNA-XR_377380.2 was increased in the retina of FDM mice (homologue of guinea pig lncRNA-XR_002792574.1) (*p* < 0.05) (Fig. 6F). We further measured the expression of the first three mRNAs closely related to lncRNA-XR_002792574.1 in the ceRNA analysis, and the result of Adcy1 expression was consistent with the RNA sequencing data analysis results in FDM guinea pigs (Fig. 4L). As expected, the expression trends of Adcy1 mRNA in the retinas of FDM mice and highly myopic adults were consistent with those in FDM guinea pigs (Figs. 6G, 8A). Moreover, immunohistochemistry analysis confirmed that Adcy1 expression in the GCL in FDM guinea pigs was increased (Fig. 4M, N). Immunofluorescence results also further confirmed that Adcy1 expression was elevated in the RCGs of FDM mice and highly myopic adults (Figs. 6I, J, 8M).

Adenylate cyclase type 1 (Adcy1) is a nerve-specific protein that catalyzes the production of cAMP and preferentially accumulates at the postsynaptic density [45, 46]. Studies have shown that an increase in cAMP accumulation can cause scleral remodeling and promote the progression of myopia [47]. In mice overexpressing adcy1, the levels of p-ERK in the forebrain are elevated above normal [48]. Elevated expression of p-ERK aggravates scleral remodeling in myopia [49]. Because adcy1 activity can be dynamically regulated by calcium and neuronal stimulation, adcy1 function is related to the regulation of neuronal signal transduction [50]. A report has also pointed out that adcy1 overexpression causes abnormal neuronal signal transduction [51]. Therefore, an increase in adcy1 may be closely related to the pathogenesis of myopia, and it may also be associated with the abnormal signal transduction of myopic RGCs. However, how adcy1 regulates myopia progression remains unknown.

Finally, we verified the first four miRNAs that may interact with both lncRNA-XR_002792574.1 and Adcy1 mRNA in the ceRNA analysis (Fig. 4F–I), and miR-760-3p expression was downregulated in FDM guinea pig RGCs (Fig. 4H). As we expected, the expression trends of miR-760-3p in the retinas of FDM mice and highly myopic adults were consistent with those in FDM guinea pigs (Figs. 6H, 8B). Recent studies have shown that miR-760-3p inhibits neuronal ferroptosis by targeting CHAC1 in neurons [52]. However, whether miR-760-3p in RGCs is associated with myopia remains unknown. We further predicted that lncRNA-XR_002792574.1 can target miR-760-3p (Fig. 4O) and that miR-760-3p can target Adcy1 through the Bibiserv database (Figs. 4P, 6E, 8C). Altogether, these results indicate that lncRNA-XR_002792574.1 may be involved in the development of myopia through the miR-760-3p/Adcy1 pathway in RGCs.

We further investigated the biological functions of lncRNAs in ceRNA networks. The GO analysis showed that the terms were enriched in calcium channel regulator activity and GABA­ergic synapse (Fig. 4A). T-type Ca^2+^ channel activation contributes to RGC apoptosis in glaucoma [53], and the numbers of GABAergic amacrine cells in the retinas of FDM guinea pigs are significantly reduced [23]. KEGG enrichment analysis showed that the main pathways included the calcium signaling pathway, MAPK signaling pathway and cAMP signaling pathway (Fig. 4B). Imbalances in the calcium signaling pathway and MAPK signaling pathway may be two key reasons for the pathological growth of lenses in patients with high myopia [54], and cAMP is involved in myopia pathogenesis [47]. The above findings are consistent with the results of our bioinformatics analysis.

Importantly, the Adcy1 mRNA downstream of lncRNA-XR_002792574.1 was closely related to the cGMP/PKG and apelin signaling pathways according to KEGG analysis (Fig. 4B). Clinical studies have shown that the cGMP/PKG signaling pathway is one of the metabolic pathways in intraocular fluid that is involved in pathological myopia [55]. It has also been reported that specific G protein-coupled receptor agonists can reduce T-type Ca^2+^ currents in rat RGCs through the NO/cGMP/PKG signaling pathway, thereby protecting RGCs from damage [40]. The sGC/cGMP/PKG pathway plays a key role in the refinement of connections in the developing retinal protogenetic pathway [56]. In addition, apelin is an endogenous oligopeptide ligand of the G protein-coupled receptor APJ [57]. Activation of the apelin receptor prevents NMDA-induced retinal neuronal cell loss in mice [58], and the apelin/APJ system can inhibit the loss of RGCs and protect the optic nerve from injury [59]. Another report has suggested that endogenous apelin can prevent age-associated loss of RGCs in mice [60]. However, it is still unknown whether apelin is associated with RGC damage in myopia.

**Fig. 7:**
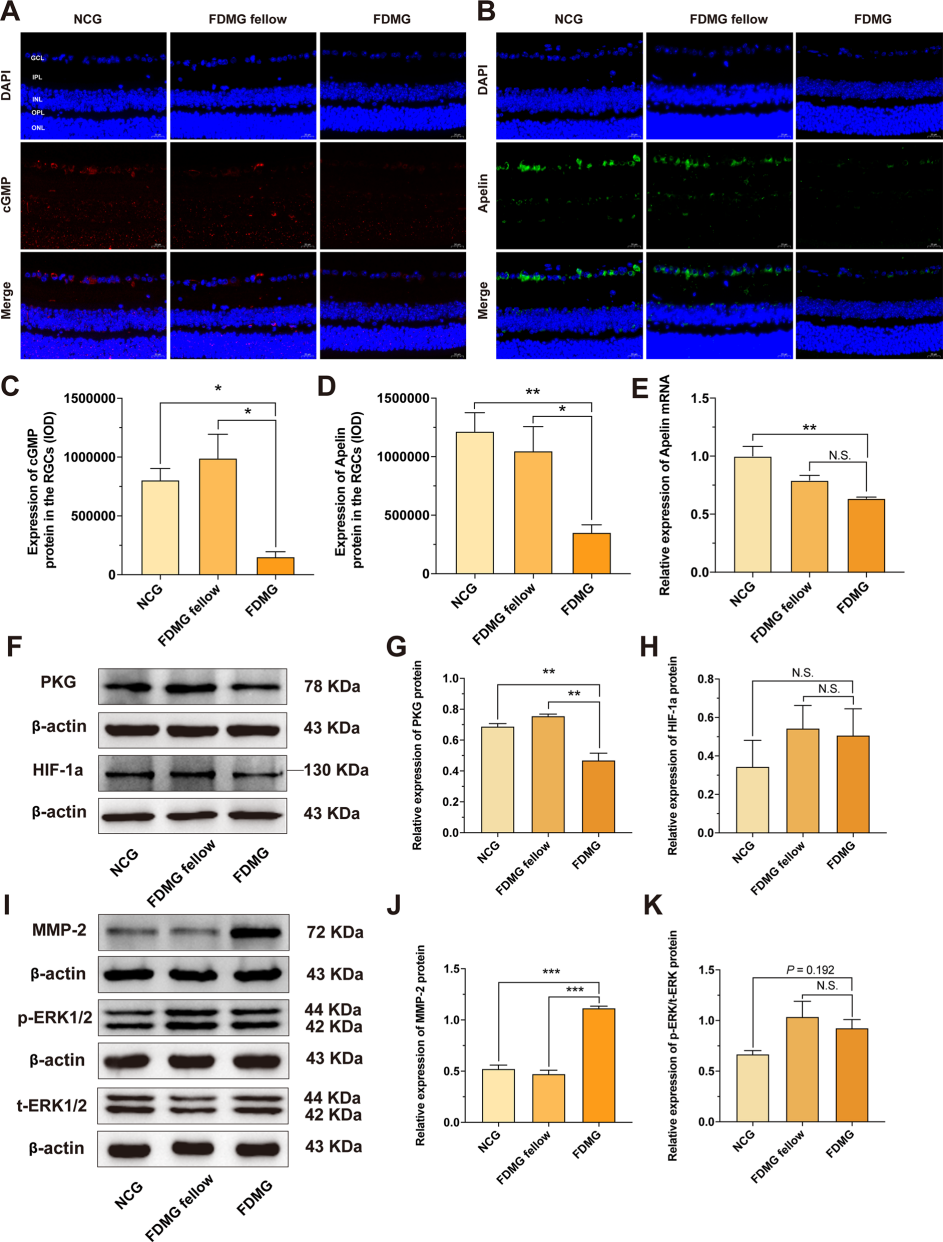
Further validation of potential regulatory cGMP/PKG and Apelin pathways of miR-760-3p/Adcy1 in FDM mouse retinas its potential relationship with sclera remodeling. **A** Images of cGMP (red) immunofluorescence in retina from mice with nucleus (blue) (scale bar: 20 µm). **B** Images of Apelin (green) immunofluorescence in retina from mice with nucleus (blue) (scale bar: 20 µm). **C**, **D** IOD analysis of cGMP expression (**C**) and Apelin (**D**) in RGCs from mice. (**E**) The relative expressions of Apelin mRNA in retina were measured by qRT-PCR. **F**–**H** Western blot analysis of PKG protein expression (**F**, **G**) and HIF-1a protein expression (**F**, **H**) in mouse retinas. **I**–**K** Western blot analysis of MMP-2 protein expression (**I**, **J**) and p-ERK/t-ERK protein expression (**I**, **K**) in sclera from mice. For each group, n = 3–5. ^*^P < 0.05, ^**^P < 0.01, ^***^P < 0.001. NCG: normal control group; FDMG fellow: form-deprived myopia fellow control group; FDMG: form-deprived myopia group

Therefore, we examined the cGMP/PKG and apelin signaling pathways in RGCs. The results showed that the cGMP/PKG and apelin signaling pathways were inhibited in the RGCs of FDM guinea pigs (Fig. 5A–F). Consistent with the results in FDM guinea pigs, the cGMP/PKG and apelin signaling pathways were also inhibited in the retinas of FDM mice and highly myopic adults (Figs. 7A–G) 8D–F, L, N). The results from FDM guinea pigs, FDM mice and highly myopic adults indicated that the lncRNA-XR_002792574.1/miR-760-3p/Adcy1 axis might have a potential relationship with the cGMP/PKG and apelin pathways and that lncRNA-XR_002792574.1 might inhibit the cGMP/PKG and apelin signaling pathways in RGCs through miR-760-3p/Adcy1, thereby causing RGC damage in myopia.

**Fig. 8:**
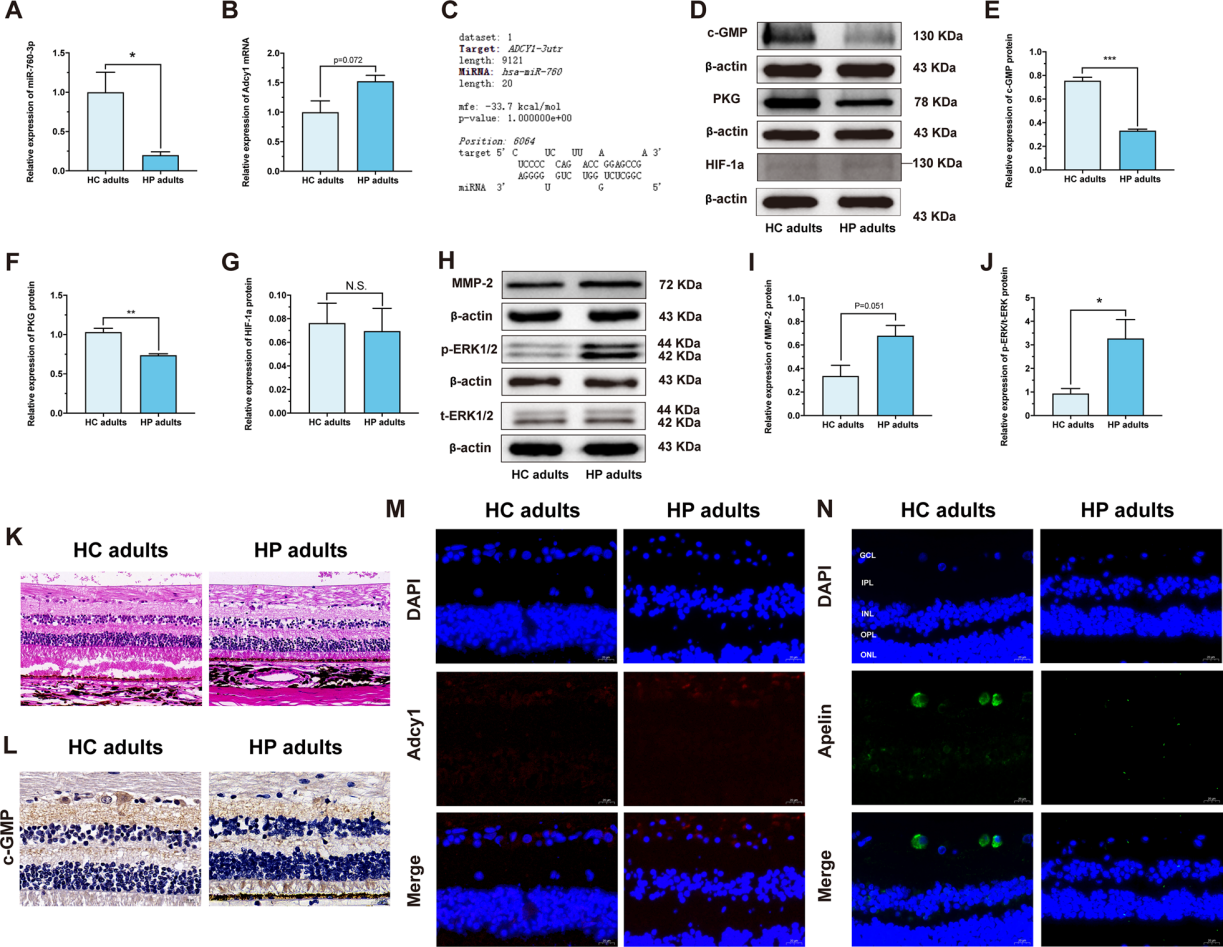
Further validation of miR-760-3p/Adcy1 and related cGMP/PKG and Apelin pathways in adult retinas with high myopia and their potential relationship with sclera remodeling. **A** The relative expressions of miR-760-3p were measured by qRT-PCR in human retinas (n = 3). **B** The relative expressions of Adcy1 mRNA were measured by qRT-PCR in human retinas (n = 3). **C** The targeting relationship between miR-760 and Adcy1 was predicted. **D**–**G** Western blot analysis of cGMP protein expression (**D**, **E**), PKG protein expression (**D**, **F**) and HIF-1a protein expression (D, G) in human retinas (n = 3). **H**–**J** Western blot analysis of MMP-2 protein expression (**H**, **I**) and p-ERK/t-ERK protein expression (**H**, **J**) in sclera from human (n = 3). **K** Morphological observation with H&E-stained retinas in human (scale bar: 50 µm). **L** Images of c-GMP (brown) immunohistochemistry in retina from human (scale bar: 20 µm). **M** Images of Adcy1 (red) immunofluorescence in retina from human with nucleus (blue) (scale bar: 20 µm). **N** Images of Apelin (green) immunofluorescence in retina from human with nucleus (blue) (scale bar: 20 µm). ^*^P < 0.05, ^**^P < 0.01, ^***^P < 0.001. HC adults: healthy control adults; HP adults: high myopia adults

Recent studies have found that local scleral hypoxia plays an important role in the development of myopia [34, 61]. However, we found that there were no changes in HIF-1α levels in the retinas of FDM guinea pigs (Fig. 5E, G). Consistent with the results in guinea pigs, the levels of HIF-1a in the retinas of FDM mice and highly myopic adults did not change (Figs. 7F, H, 8D, G). This suggests that myopia may not cause retinal hypoxia, only local scleral hypoxia. Scleral remodeling is known to be closely related to myopia development [35, 36]. Studies have shown that phosphorylation of ERK1/2 leads to the upregulation of MMP-2 transcription and translation, which then regulates ECM degradation and causes scleral remodeling in FDM guinea pigs [49]. It was recently reported that ADCY1 activation stimulates ERK phosphorylation [48, 62]. In this study, we also found that MMP-2 and p-ERK/t-ERK protein expression in the sclerae of FDM guinea pigs, FDM mice and highly myopic adults tended to increase (Figs. 5H–J, 7I–K, 8H–J). The results indicated that the lncRNA-XR_002792574.1/miR-760-3p/Adcy1 axis might cause scleral remodeling in FDM guinea pigs.

Therefore, we demonstrated that the lncRNA-XR_002792574.1/miR-760-3p/Adcy1 axis in RGCs might be related to myopia. However, potential limitations should be mentioned in this study. We only predicted the LncRNA-XR_002792574.1-mediated ceRNA network and related regulatory pathways via bioinformatics analysis and molecular experiments in FDM guinea pigs, FDM mice, and highly myopic adults. These findings may reveal novel potential targets for myopia treatment, but it is not yet a solid alternative to existing therapeutics. In future myopia studies, we plan to further verify whether inhibiting lncRNA-XR_002792574.1 regulates the cGMP/PKG and apelin signaling pathways in RGCs and scleral remodeling via miR-760-3p/Adcy1, thereby delaying myopia progression. Compared with the traditional gene delivery vector of adeno-associated viruses [63], green nanomaterials have the advantages of non-toxicity, low cost and antibacterial properties [64, 65]. Therefore, we may consider trying to use green nanomaterials to encapsulate gene overexpression/inhibitors to target the retina for myopia treatment in the future. In addition, further studies with a large clinical sample sizes are needed to elucidate the role of LncRNA-XR_002792574.1-mediated ceRNA network in myopia.

## Conclusion

**Fig. 9:**
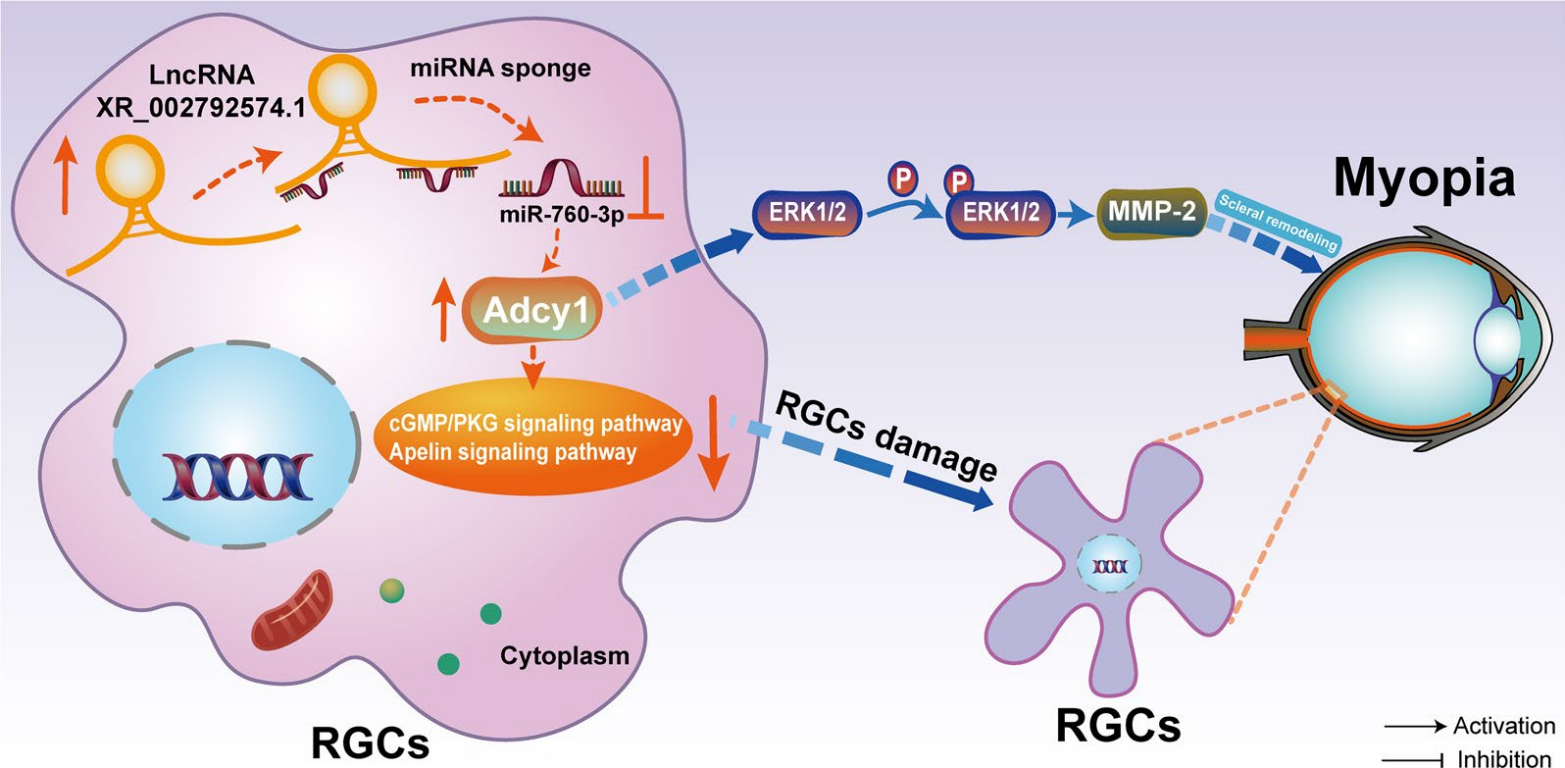
Schematic diagram illustrating the mechanism. lncRNA-XR_002792574.1 may be involved in the development of myopia through miR-760-3p/Adcy1 pathway in RGCs. On the one hand, lncRNA-XR_002792574.1 might inhibited the cGMP/PKG and Apelin signaling pathways in RGCs through miR-760-3p/Adcy1, thereby causing RGCs damage in myopia. On the other hand, lncRNA-XR_002792574.1/miR-760-3p/Adcy1 axis may cause myopic scleral remodeling through the ERK-MMP-2 pathway. RGCs: retinal ganglion cells

In summary, we identified the expression profiles of lncRNAs and mRNAs and constructed a ceRNA network in the RGCs of FDM guinea pigs. Given further verification in FDM guinea pigs, FDM mice, and highly myopic adults, we propose that lncRNA-XR_002792574.1 may be involved in the development of myopia through the miR-760-3p/Adcy1 pathway in RGCs (Fig. 9). In addition, we found that the lncRNA-XR_002792574.1/miR-760-3p/Adcy1 axis in RGCs might be related to cGMP/PKG, the apelin signaling pathway and scleral remodeling in FDM guinea pigs, FDM mice, and highly myopic adults (Fig. 9). On the one hand, the lncRNA-XR_002792574.1/miR-760-3p/Adcy1 axis might inhibit the cGMP/PKG and apelin signaling pathways in RGCs, thereby causing RGC damage in myopia. On the other hand, the lncRNA-XR_002792574.1/miR-760-3p/Adcy1 axis may cause myopic scleral remodeling through the ERK-MMP-2 pathway (Fig. 9). Overall, regulating cGMP/PKG and apelin signaling in RGCs and scleral remodeling by inhibiting lncRNA-XR_002792574.1 may be a candidate strategy for preventing myopia progression, which provides a favorable reference value for subsequent exploration and development of gene editing therapeutics for hereditary myopia.

### Supplementary Information

**Additional file 1:** **Table S1.** Primer sequences of guinea pig. **Table S2.** Primer sequences of mice. **Table S3.** Primer sequences of human. **Table S4.** Top 10 upregulated and 10 downregulated DE lncRNAs in FDMG guinea pigs compared to NCG guinea pigs. **Table S5.** Top 10 upregulated and 10 downregulated DE mRNAs in FDMG guinea pigs compared to NCG guinea pigs. **Figure S1.** GO and KEGG enrichment analysis of the DE mRNAs between FDMG and NCG guinea pig. **Figure S2.** LncRNA-mRNA network analysis.

## Data Availability

The analyzed datasets generated during the study are available from the cor-responding author upon reasonable request.

## Abbreviations

lncRNA: Long non-coding RNA
miRNA/miR: MicroRNA
RGCs: Retinal ganglion cells
ceRNA: Competing endogenous RNA
FDM: Form-deprived myopia
LIM: Lens-induced myopia
KEGG: Kyoto Encyclopedia of Genes and Genomes
ECM: Extracellular matrix
ERK: Extracellular signal‐regulated kinase
MAPK: Mitogen‐activated protein kinase
PBS: Phosphate‐buffered saline
qRT-PCR: Quantitative real time polymerase chain reaction
OCT: Optical coherence tomography
H&E: Hematoxylin and Eosin
WB: Western blot
Adcy1: Adenylate cyclase type 1
cGMP: Cyclic guanosine monophosphate
PKG: Protein kinase G
