# Supplementary material for: LncRNA-XR_002792574.1-mediated ceRNA network reveals potential biomarkers in myopia-induced retinal ganglion cell damage
Source: J Transl Med. 2023 Nov 6;21:785. doi: 10.1186/s12967-023-04662-x (PMC10629108; doi:10.1186/s12967-023-04662-x)
Supplement: Supplementary file 1 — Additional file 1: Table S1. Primer sequences of guinea pig. Table S2. Primer sequences of mice. Table S3. Primer sequences of human. Table S4. Top 10 upregulated and 10 downregulated DE lncRNAs in FDMG guinea pigs compared to NCG guinea pigs. Table S5. Top 10 upregulated and 10 downregulated DE mRNAs in FDMG guinea pigs compared to NCG guinea pigs. Figure S1. GO and KEGG enrichment analysis of the DE mRNAs between FDMG and NCG guinea pig. Figure S2. LncRNA-mRNA network analysis. [file 12967_2023_4662_MOESM1_ESM.docx]

**Supplementary materials**

*Supplementary Table 1 primer sequences of guinea pig*

| Gene | Primer F | Primer R |
| --- | --- | --- |
| *Cpo-XR_001202354.2* | 5'CTGCTCACCAAGATGCGTTC 3' | 5'CCACCATGCAGAGACTTGGA 3' |
| *Cpo-XR_002792574.1* | 5'TTCGTTCCGTGAACCATCCC 3' | 5'AGCCTCTCCCAAGAAGGTGA 3' |
| *Cpo-XR_001199906.2* | 5'GATGCCCAGAGACCACAGTT 3' | 5'TGGAGCAAGCAAACCTCGTA 3' |
| *Cpo-miR-532-3p* | 5'GACGCCTCCCACACCCAAG 3' | - |
| *Cpo**-miR-342-5p* | 5'AGGGGTGCTATCTGTGCTGG3' |  |
| *Cpo-miR-760-3p* | 5'CTCGGCTCTGGGTCTGTGG3' |  |
| *Cpo-miR-574-5p* | 5'AACACGTGTGAGTGTGTGTGTG3' |  |
| *Cpo-Adcy1* | 5'TGACGAGTTAGCCACGGAGA3' | 5' ACCACACGTCATACTGCCAC 3' |
| *Cpo-Elfn2* | 5'TGGAGAGGAAACTGACACG  C3' | 5'CCTTACGACCACATCCCCAC3' |
| *Cpo-Xkr7* | 5'CTTGCCTCCCACACCAGTAG3' | 5'AGTGCCAGAATGGTCTTCCG3' |
| *Cpo-Gapdh* | 5'GCTGATGCCCCTATGTTCGT 3' | 5'GGATGCGGGGATGATGTTCT3' |
| *U6* | 5' CCTGCTTCGGCAGCACA3 3' | - |

*Supplementary Table 2 primer sequences of mice*

| Gene | Primer F | Primer R |
| --- | --- | --- |
| *Mmu-XR_377380.2* | 5'TCAGTGGGCACCGTGATTCT3' | 5'AAGAGGTCAATGAGGGCACATC 3' |
| *Mmu-miR-760-3p* | 5'AACAAGCGGCTCTGGGTCT3' | - |
| *Mmu-Adcy1* | 5'TTGCCTCCATCCCCAACTTC3' | 5'TGGACTTCTTAGCCCTGGTTC3' |
| *Mmu-Apelin* | 5'CCTCTAATGGCGTGGTCTCG3' | 5'GTCTCCAAGGGCAGTCCAAA3' |
| *Mmu-Gapdh* | 5'AGGTCGGTGTGAACGGATTTG 3' | 5'TGTAGACCATGTAGTTGAGGTCA3' |
| *U6* | 5' CCTGCTTCGGCAGCACA3 3' | - |

*Supplementary Table 3 primer sequences of human*

| Gene | Primer F | Primer R |
| --- | --- | --- |
| *hsa-miR-760-3p* | 5'AACGATACGGCTCTGGGTCTG3' | - |
| *hsa-Adcy1* | 5'AGGCACGACAATGTGAGCATC3' | 5'TTCATCGAACTTGCCGAAGAG 3' |
| *hsa-Gapdh* | 5' AAAATCAAGTGGGGCGATGC 3' | 5' TGGTTCACACCCATGACGAA 3' |
| *U6* | 5' CCTGCTTCGGCAGCACA3 3' | - |

*Supplementary Table 4 top 10 upregulated and 10 downregulated DE lncRNAs* *in* *FDMG* *guinea pigs compared to NCG guinea pigs.*

| Gene ID | Gene symbol | Fold Change | Log2 FC | *P* | Trend |
| --- | --- | --- | --- | --- | --- |
| XR_002788881.1 | - | 2958362.173 | 21.49636725 | 3.80E-08 | UP |
| TCONS_00049891 | XLOC_035136 | 2478055.409 | 21.24077702 | 5.53E-08 | UP |
| TCONS_00043360 | - | 782.2668776 | 9.61151707 | 0.000331089 | UP |
| XR_001200558.2 | LOC106026962 | 293.9579541 | 8.199466006 | 0.005839609 | UP |
| TCONS_00055113 | XLOC_039045 | 292.7357413 | 8.193455091 | 0.001998541 | UP |
| TCONS_00029735 | XLOC_020600 | 271.8282673 | 8.086551679 | 1.28E-05 | UP |
| XR_001199029.1 | LOC106025442 | 251.2469271 | 7.972962141 | 0.000543998 | UP |
| TCONS_00057358 | XLOC_040558 | 230.7886809 | 7.850428658 | 0.012264637 | UP |
| XR_001202431.1 | LOC106028810 | 209.2759467 | 7.709262693 | 0.006973931 | UP |
| XR_002790391.1 | LOC101788686 | 145.1308758 | 7.181210668 | 0.043307208 | UP |
| TCONS_00048275 | XLOC_033964 | 4.82E-07 | -20.9849343 | 7.97E-08 | DOWN |
| XR_002790344.1 | - | 0.003741817 | -8.0620452 | 3.08E-10 | DOWN |
| XR_002790991.1 | LOC106027513 | 0.00485601 | -7.6860128 | 0.007777034 | DOWN |
| XR_002789406.1 | LOC106025878 | 0.008633589 | -6.8558239 | 0.002760949 | DOWN |
| XR_001202662.2 | LOC106029027 | 0.025603539 | -5.2875129 | 0.039735913 | DOWN |
| XR_002790363.1 | LOC106026912 | 0.026936001 | -5.2143205 | 0.048377552 | DOWN DOWN |
| TCONS_00079558 | XLOC_056129 | 0.039102884 | -4.6765811 | 0.000816273 | DOWN |
| XR_001200351.2 | LOC106026777 | 0.069079962 | -3.8555889 | 0.003834242 | DOWN |
| XR_001202674.2 | LOC106029033 | 0.095371368 | -3.3902999 | 0.000720109 | DOWN |
| XR_001202682.2 | - | 0.096662458 | -3.3709005 | 5.07E-05 | DOWN |
|  |  |  |  |  |  |

DE: differentially expressed; FC: fold change.

*Supplementary Table 5 top 10 upregulated and 10 downregulated DE mRNAs in FDMG guinea pigs compared to NCG guinea pigs.*

| Gene ID | Fold Change | Log2 FC | *P Value* | Trend |
| --- | --- | --- | --- | --- |
| Trpc4 | 62.51176816 | 5.966055905 | 4.52E-08 | UP |
| Opn1mw | 58.43018442 | 5.868641938 | 1.63E-42 | UP |
| Cartpt | 34.52881857 | 5.109729067 | 1.52E-13 | UP |
| B4galnt1 | 34.01253735 | 5.087994731 | 7.42E-07 | UP |
| Cpne6 | 33.30624665 | 5.057720878 | 1.16E-30 | UP |
| Calb1 | 30.91423218 | 4.950199269 | 2.69E-10 | UP |
| Gng13 | 30.3836271 | 4.9252222 | 1.43E-13 | UP |
| Gabra1 | 29.5974941 | 4.88740313 | 4.16E-22 | UP |
| Tex28 | 27.4068398 | 4.77646408 | 2.82E-23 | UP |
| Tmem196 | 26.5765056 | 4.73207952 | 1.21E-05 | UP |
| Tnnt3 | 0.01138771 | -6.4563786 | 2.77E-06 | DOWN |
| Atp2a1 | 0.03433715 | -4.8640857 | 0.00102852 | DOWN |
| LOC100727418 | 0.03993284 | -4.6462805  2 | 3.92E-08 | DOWN |
| Ckm | 0.04262427  1 | -4.552181 | 2.52E-05 | DOWN |
| Cryga | 0.05060207 | -4.3046598 | 0.00028567 | DOWN |
| Myh4 | 0.07279972 | -3.7799233 | 1.93E-08 | DOWN DOWN |
| LOC100727012 | 0.07374231 | -3.7613635 | 1.85E-05 | DOWN |
| Ca3 | 0.10923852 | -3.1944464 | 0.00637659 | DOWN |
| Pkd1l2 | 0.11032358 | -3.1801869 | 1.01E-12 | DOWN |
| Elf5 | 0.11658075 | -3.1005986 | 0.00037824 | DOWN |
|  |  |  |  |  |

DE: differentially expressed.

*Supplementary Fig. 1* GO and KEGG enrichment analysis of the DE mRNAs between FDMG and NCG guinea pig


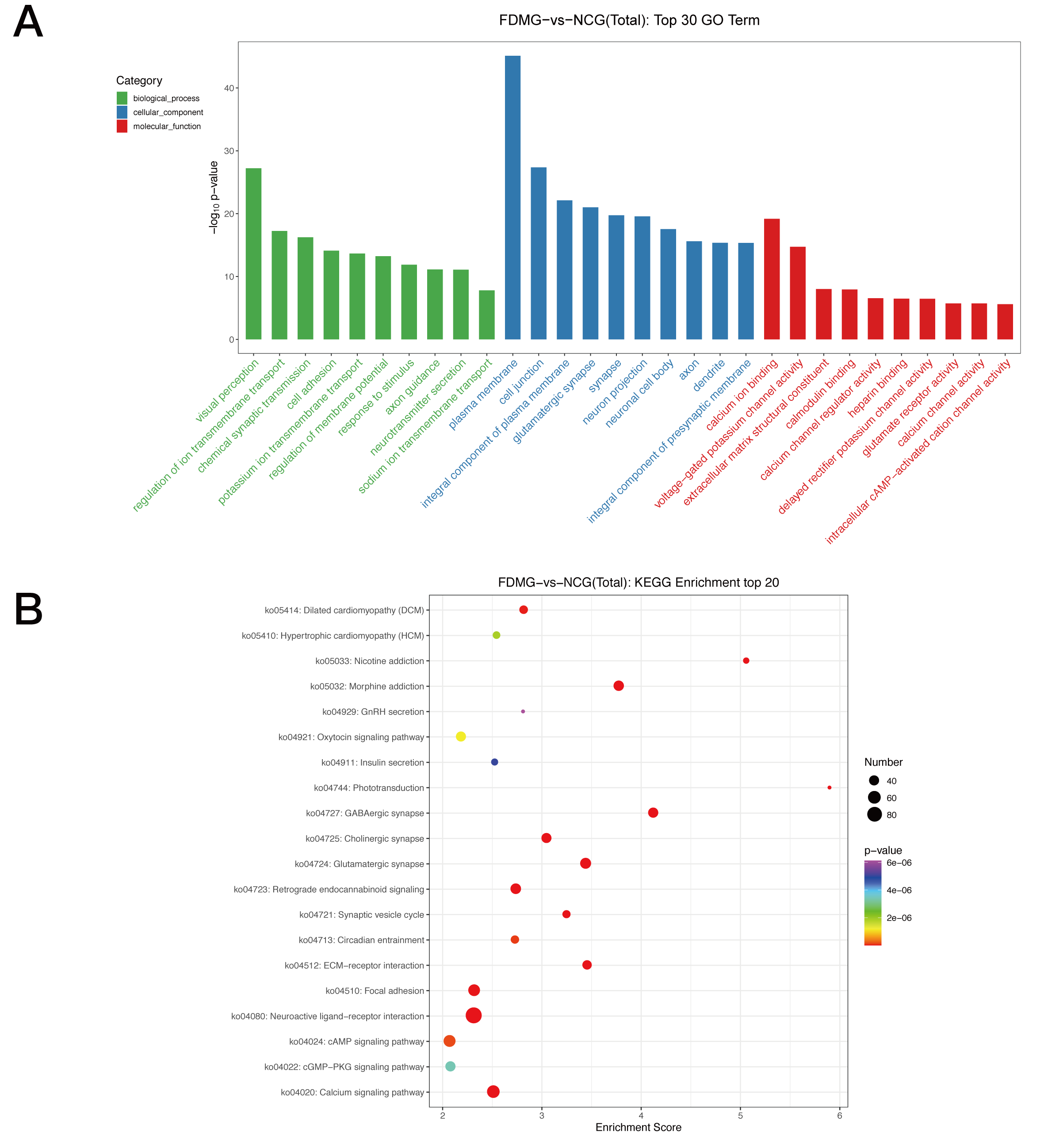


Supplementary Fig. 1 (A) top 30 GO terms from the mRNA enrichment analysis and (B) top 20 significant KEGG pathways between FDMG and NCG. DE: differentially expressed; NCG: normal control group; FDMG: form-deprived myopia group.

*Supplementary Fig. 2* LncRNA-mRNA network analysis.


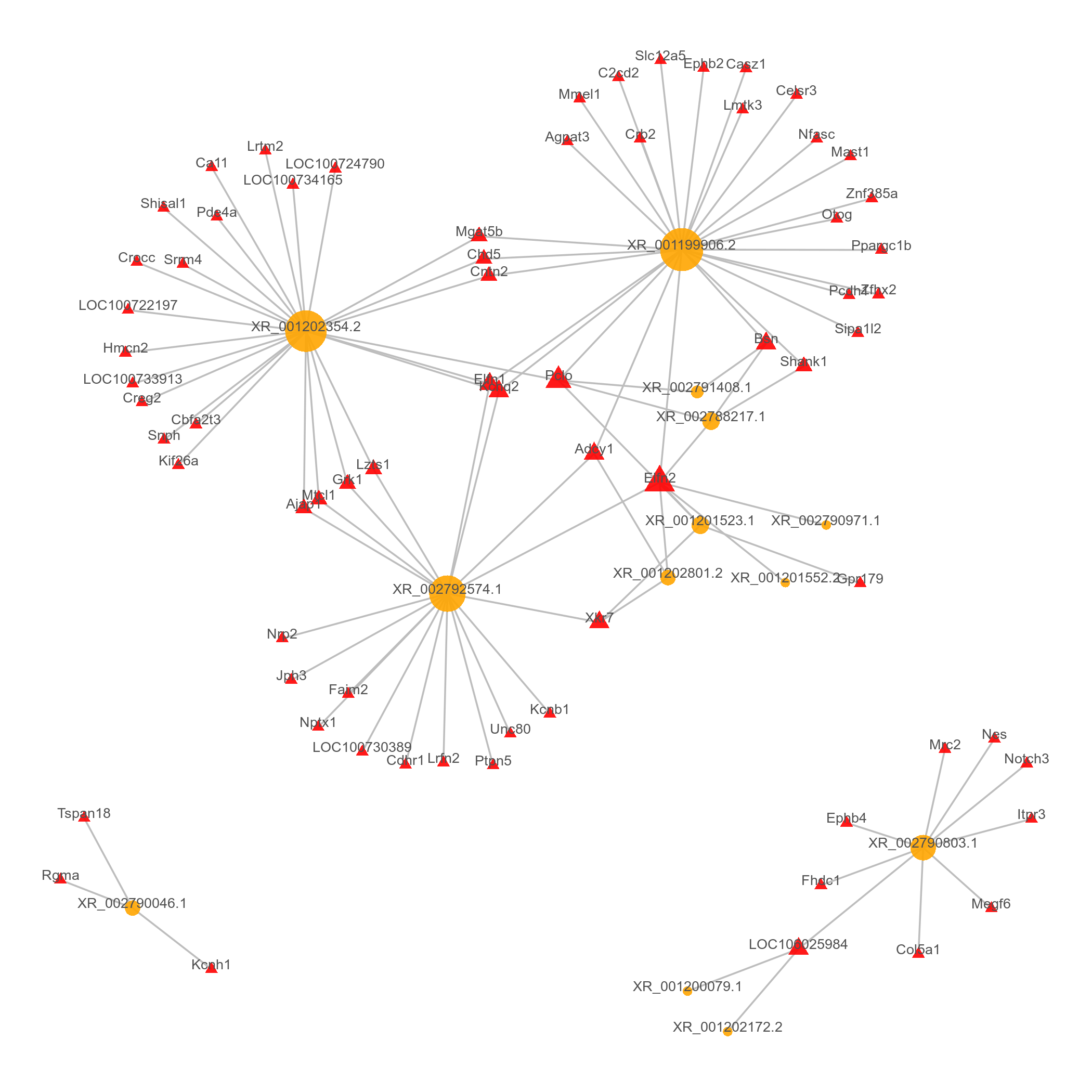


Supplementary Fig. 2 Top100 LncRNA-mRNA network analysis. orange and red represent lncRNAs and mRNAs, respectively.
